# Natural Variation in Wheat Homologs of the Rice Thermotolerance Gene *QT12* Reveals Two Divergent Haplotypes of *TaQT12-B1*

**DOI:** 10.3390/plants15213345

**Published:** 2026-10-28

**Authors:** Mikhail S. Bazhenov, Timofey D. Mokhov, Yana S. Meglitskaya, Kristina O. Petrova, Ludmila A. Bespalova, Nadezhda A. Polevikova, Mikhail G. Divashuk

**Affiliations:** 1 All-Russia Research Institute of Agricultural Biotechnology, 42 Timiryazevskaya Street, Moscow 127550, Russia; timmokh@yandex.ru (T.D.M.); yanameg20@gmail.com (Y.S.M.); divashuk@gmail.com (M.G.D.); 2 National Research Center “Kurchatov Institute”, 1 Akademika Kurchatova Square, Moscow 123098, Russia; petrova.k.o@yandex.ru; 3 Department of Breeding and Seed Production of Wheat and Triticale, National Center of Grain Named After P.P. Lukyanenko, Central Estate of KNIISH, Krasnodar 350012, Russia; bespalova_l_a@rambler.ru (L.A.B.); polevika94@mail.ru (N.A.P.)

**Keywords:** heat stress, Sec61β, pangenome, KASP marker, cis-regulatory polymorphism, grain yield, grain quality, Oxford Nanopore sequencing

## Abstract

The rice gene *QT12*, which encodes the β subunit of the Sec61 translocon complex, has previously been shown to negatively regulate grain thermotolerance, suggesting that variation in related cereal genes may contribute to heat responses. We characterized the natural sequence diversity of three *QT12* homeologs located on homoeologous group 3 chromosomes in bread wheat using publicly available whole-genome assemblies and Oxford Nanopore amplicon sequencing. *TaQT12-A1* and *TaQT12-D1* were highly conserved in their transcribed regions, whereas *TaQT12-B1* was represented by two strongly differentiated haplotype groups, Hap1 and Hap2, distinguished by 108 sequence variants across the gene and its 7-kb flanking regions. The protein-coding sequence of *TaQT12-B1* was conserved between the two haplotype groups, while most differences occurred in non-coding and flanking regions. A KASP marker was developed to distinguish the two groups and used to genotype 200 winter wheat accessions, of which six carried Hap2. Comparisons of grain-related traits over 2018–2020 revealed differences between haplotypes, but none remained significant after false discovery rate correction. Seasonal temperature records provided environmental context but did not support a clear haplotype-specific heat-response pattern. These results identify *TaQT12-B1* as a candidate locus with substantial non-coding sequence variation and provide a molecular marker for further functional investigation of its haplotypes.

## 1. Introduction

Rising temperatures associated with climate change represent a major threat to wheat production worldwide. Modeling and multi-method assessments indicate that global wheat yield may decline by approximately 6% for each 1 °C increase in mean temperature in the absence of effective adaptation and genetic improvement [1,2]. The development of wheat cultivars capable of maintaining grain yield and quality under episodes of high temperature is therefore an important objective for crop improvement.

The effects of heat stress strongly depend on the developmental stage at which it occurs. Reproductive development and grain filling are among the most temperature-sensitive phases in wheat [3,4]. At early booting, when male meiosis and microsporogenesis take place, short episodes of high temperature can disrupt meiotic progression and subsequently reduce grain number [5,6]. High temperature around flowering can impair reproductive processes and reduce grain set, whereas post-anthesis heat accelerates grain development, shortens the grain-filling period, and commonly reduces individual grain weight and final yield [4,7]. Heat during grain filling also affects starch accumulation and grain protein composition and can consequently alter technological and nutritional grain quality [8,9].

**Figure 1:**
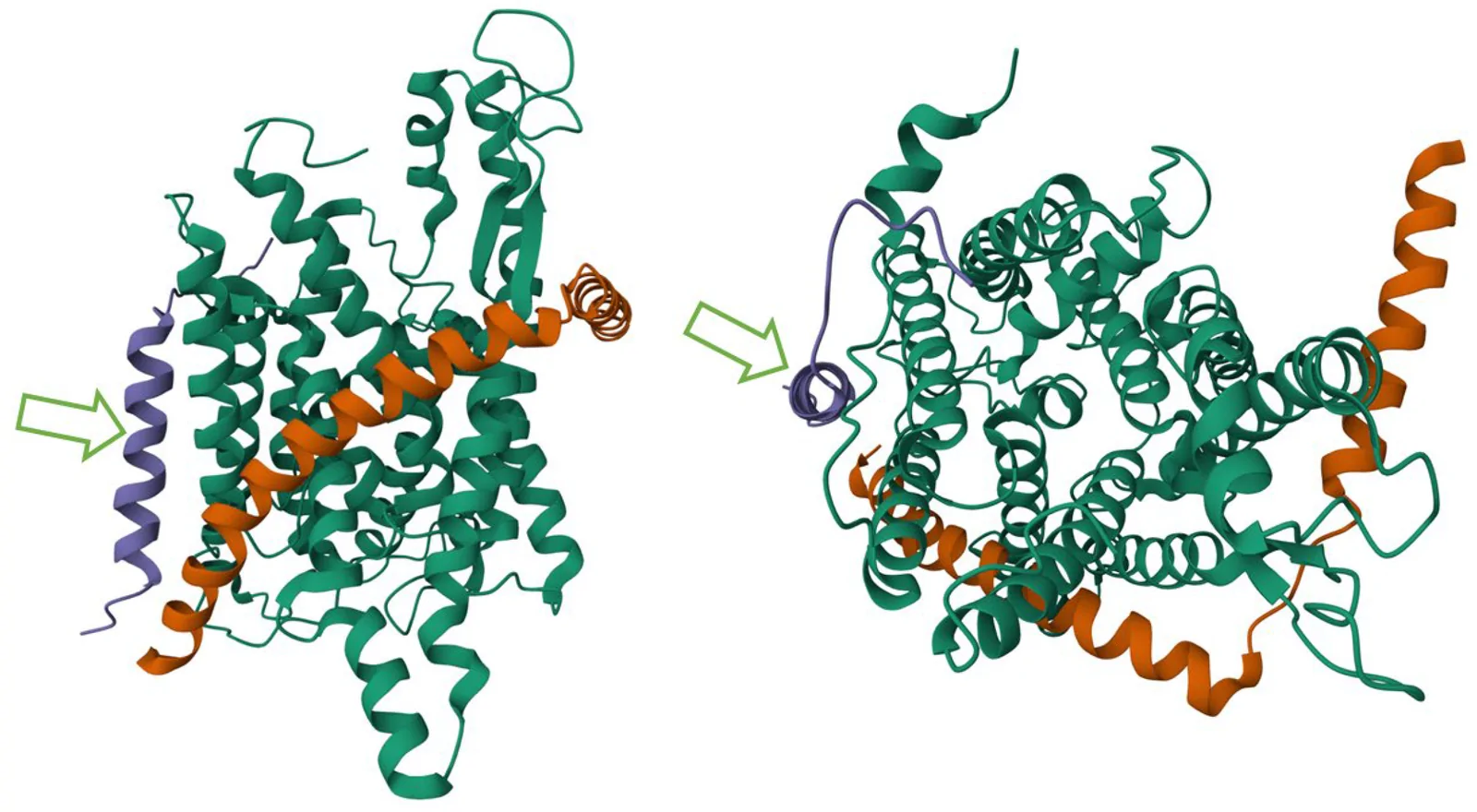
Three-dimensional structure of the protein-conducting channel from the thermophilic methanogenic archaeon *Methanocaldococcus jannaschii*. SecY(α) is shown in green, SecE(γ) in orange, and Secβ(β) in blue-violet (arrow). The homologous β subunit of the eukaryotic Sec61 complex is encoded by *QT12* in rice. Structure: PDB entry 1RHZ [11].

Recent findings in rice have identified *QT12* as a molecular component linking high-temperature exposure with grain quality and yield. *QT12* encodes the β subunit of the Sec61 translocon (Figure 1) and acts as a negative regulator of grain-quality thermotolerance. Under high temperature, increased *QT12* expression causes excessive endoplasmic-reticulum stress in the developing endosperm, interferes with the normal accumulation of starch and storage proteins, and ultimately increases grain chalkiness in rice. This effect was associated with overactivation of the unfolded protein response, a cellular pathway activated by protein-folding stress in the endoplasmic reticulum. Natural variation in the *QT12* promoter affects its transcriptional regulation by an NF-Y transcription factor complex, while alleles associated with reduced *QT12* expression or loss of function improve the maintenance of grain quality and can increase yield under high-temperature field conditions [10]. These findings suggest that natural regulatory variation in *QT12*-related genes may represent a potentially important component of heat response in other cereal species.

The protein encoded by *QT12* belongs to the evolutionarily conserved protein-conducting translocon of the endoplasmic reticulum. The Sec61/SecY channel mediates the translocation and membrane insertion of newly synthesized proteins, thereby contributing to protein targeting and proteostasis [11,12]. For example, Sec61β homologs contribute to temperature tolerance in yeast in a species-dependent manner: loss of both *SBH1* and *SBH2* in *Saccharomyces cerevisiae* causes heat-sensitive growth and impaired ER protein translocation at elevated temperature, whereas deletion of *sbh1* in *Schizosaccharomyces pombe* results in cold sensitivity and reduced protein secretion at low temperature [13,14]. More recent work showed that Sec61β facilitates endoplasmic reticulum translocation of a subset of proteins with relatively weak targeting signals, including proteins with less hydrophobic signal sequences that are inefficiently inserted into the Sec61 channel in its absence [15]. Together with the results obtained for rice *QT12*, these observations provide a rationale for investigating natural variation in its wheat homologs.

Bread wheat (*Triticum aestivum* L.) is an allohexaploid species, and orthology data indicate a one-to-many relationship between rice *QT12* and six wheat co-orthologs organized into two homeologous triads. The chromosome group 3 triad comprises *TraesCS3A02G207800*, *TraesCS3B02G234900*, and *TraesCS3D02G208200*, whereas the second triad comprises *TraesCS5A02G015200*, *TraesCS5B02G013400*, and *TraesCS5D02G021100*. Publicly available expression profiles from the Wheat Expression Browser (expVIP; [16,17]) indicate that the chromosome group 3 triad is generally more strongly expressed across major tissues and conditions than the group 5 triad. In particular, *TraesCS3B02G234900* (*TaQT12-B1*) shows the highest mean expression among the six candidate genes in most aggregated tissue/stress categories, including grain (Figure S6). These expression patterns provided the rationale for selecting the chromosome group 3 triad for detailed sequence analysis, with particular attention to *TaQT12-B1*.

The availability of increasingly complete wheat reference genomes and multiple cultivar assemblies now makes it possible to assess natural sequence diversity of individual loci in a broader genomic context [18,19]. Comparative wheat genomics has revealed extensive cultivar-specific nucleotide and structural variation resulting from diverse breeding histories, emphasizing the potential value of haplotype-level analyses for identifying functionally relevant alleles [19,20].

In the present study, we characterized natural sequence variation in the selected chromosome group 3 *QT12* homeologs using amplicon sequencing and publicly available whole-genome assemblies, with particular emphasis on the haplotype structure of *TaQT12-B1*. We developed a KASP marker to distinguish the major *TaQT12-B1* haplotypes and assessed their distribution in a collection of 200 winter bread wheat accessions. Phenotypic comparisons across three growing seasons were treated as exploratory, and seasonal temperature records were used to provide environmental context. The primary aim was to define genomic diversity at the selected wheat *QT12* loci and determine whether *TaQT12-B1* haplotypes show phenotypic patterns that warrant further functional investigation.

## 2. Results

### 2.1. Selection and Pangenome Analysis of QT12 Homeologs

The orthology comparison identified six bread wheat co-orthologs of rice *QT12*, divided between chromosome groups 3 and 5. Expression data from the Wheat Expression Browser showed a clear difference between the two triads: the group 3 genes were generally more highly expressed, and *TraesCS3B02G234900* (*TaQT12-B1*) had the highest mean transcripts per million (TPM) among the six genes in most aggregated tissue/stress categories, including unstressed grain (Figure S6). Based on this expression pattern, subsequent pangenome and amplicon analyses focused on the chromosome group 3 homeologs.

**Figure 2:**
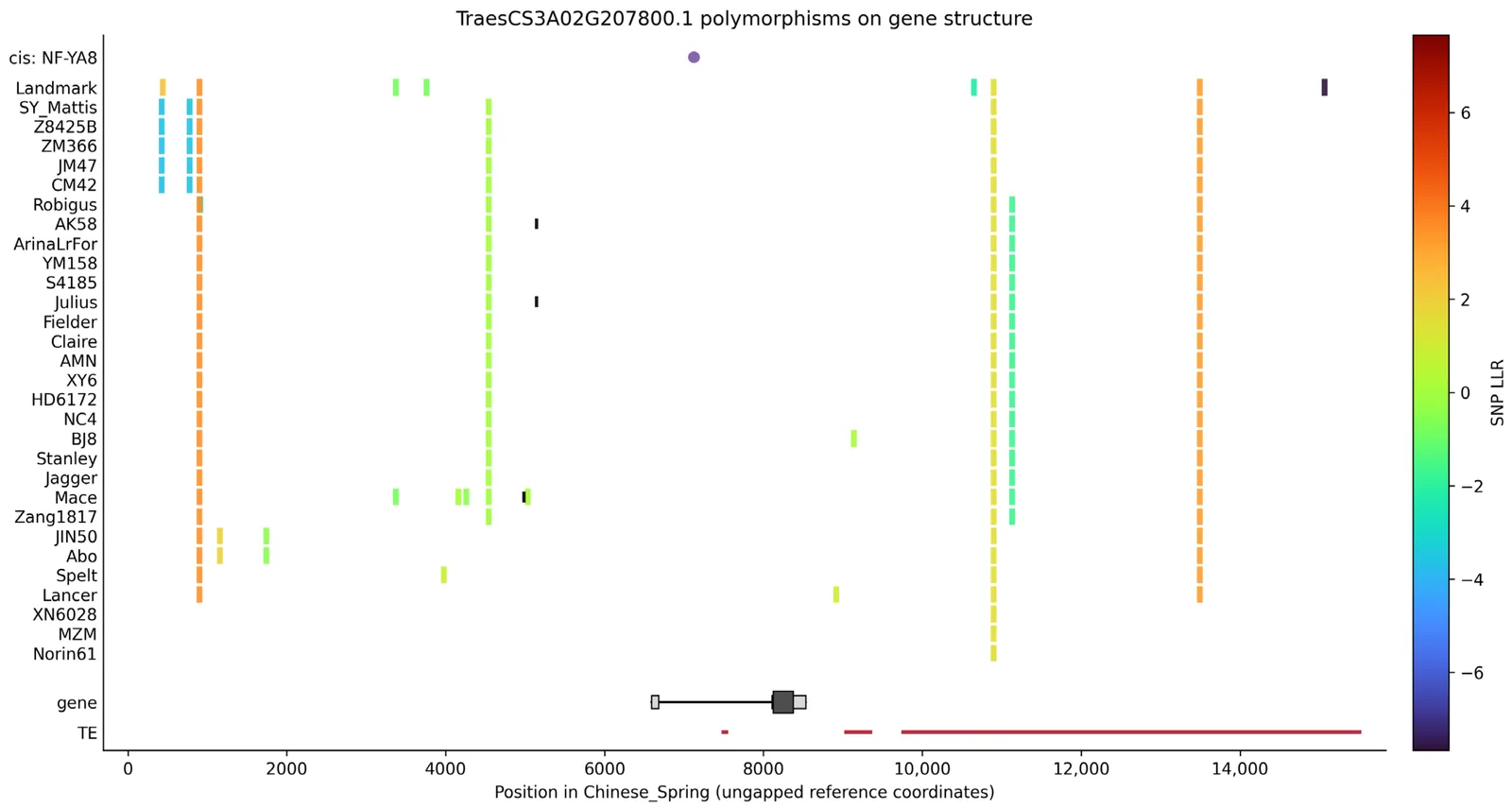
Nucleotide-sequence variation detected around *TaQT12-A1* by comparison of chromosome fragments from complete genome assemblies of hexaploid wheat accessions (bread wheat and spelt). Each accession is shown on a separate row. Single-nucleotide variants (SNVs) are represented by vertical bars colored according to their log-likelihood ratio (LLR) relative to the Chinese Spring reference allele in the local reference context (up to ±2500 bp), as predicted by the AgroNT DNA language model. Insertions are shown as thin vertical black lines, deletions as rectangular marks, and replacement variants (delins), where present, as distinct hatched rectangles. The gene model shows UTRs in light gray and CDS regions in dark gray, with introns represented by connecting lines. Predicted cis-regulatory motifs, when detected, are displayed in tracks above the accession rows, whereas annotated transposable elements are shown as red horizontal segments below the gene model. TE intervals are shown according to the Chinese Spring annotation. Transcription proceeds from left to right (5′ → 3′).

Analysis of *TaQT12-A1* variation in the hexaploid wheat pangenome, including 7000 bp of flanking chromosomal sequence on each side, showed that the TraesCS3A02G207800 gene region itself (chr3A:365964703–365966643) was highly conserved. All differences from Chinese Spring were located outside the gene, with no exonic or intronic variants detected. Of the 24 polymorphisms identified, 17 were located upstream of the transcription start site and seven downstream of the transcription termination site. Thus, sequence variation at this locus was restricted to the flanking regions and could not alter the *TaQT12-A1* protein-coding sequence (Figure 2).

A total of 21 single-nucleotide variants and three insertions were identified. Most variants were rare, although several upstream variants were common: chr3A:g.365962648T>G, located approximately 2.1 kb upstream of the gene, was detected in 22 genomes, whereas chr3A:g.365959007T>C (~5.7 kb upstream) was detected in 27 genomes. Of particular interest were three genotype-specific insertions located approximately 1.4–1.6 kb upstream of the gene in Mace, AK58, and Julius. None of these proximal upstream variants overlapped annotated transposable elements (TEs) or the cis-element sites (NF-YA8) identified by motif scanning. No miRNA-binding sites were predicted in the mature mRNA sequences of any of the three wheat *QT12* homeologs.

Zero-shot SNV scoring with the AgroNT DNA language model produced LLR values ranging from −7.66 to +3.48. The most negative score was obtained for the rare downstream variant chr3A:g.365973172G>A in Landmark (LLR = −7.66), located approximately 6.5 kb downstream of the gene. This variant also overlapped an annotated TE, which complicates interpretation of its high absolute LLR as evidence of gene-specific regulatory importance. In total, six of the 24 *TaQT12-A1* polymorphisms overlapped TEs, and all six were downstream of the gene; no upstream or intragenic variant showed TE overlap. None of the 24 polymorphisms overlapped predicted miRNA-binding sites or detected cis-element sites. Insertions were not assigned AgroNT LLR scores because the implemented masked-token zero-shot scoring procedure was restricted to SNVs (Table S4).

Analysis of *TaQT12-D1* and its flanking regions (TraesCS3D02G208200; chr3D:274727778–274728029) revealed strong conservation of the transcribed region but substantially greater variation in its flanking sequence. Of 71 unique polymorphisms, 51 were located upstream of the transcription start site, 19 downstream of the gene, and only one within the gene. The variant set comprised 56 SNVs, five insertions, seven deletions, and three delins. Mace and Robigus were particularly divergent from Chinese Spring, carrying 44 and 47 variants, respectively; 39 polymorphisms were shared exclusively by these two accessions, indicating a closely related and strongly differentiated version of the *TaQT12-D1* locus.

Most changes were rare and occurred in no more than two or three genomes. In the proximal putative promoter-regulatory region, the nearest polymorphisms occurred approximately 1.4–2.0 kb upstream of the gene. The most notable was chr3D:g.274725859A>G (~1.9 kb upstream), detected in Mace and Robigus and characterized by a strongly negative LLR (−7.29). A more distal upstream variant, chr3D:g.274724376C>G (~3.4 kb upstream), identified in Landmark and Lancer, had LLR = −9.65. Neither of these variants overlapped an annotated TE. By contrast, chr3D:g.274722806G>C (~5.0 kb upstream), present in Mace and Robigus and characterized by LLR = +8.13, was located within an annotated TE.

Overall, AgroNT scores for *TaQT12-D1* SNVs ranged from −9.96 to +12.56. The most negative variant, chr3D:g.274733869T>G in Mace (LLR = −9.96), and the most positive variant, chr3D:g.274735003C>G in AK58 (LLR = +12.56), were both located several kilobases downstream of the gene and overlapped annotated TEs. Thus, extreme AgroNT scores in distal regions should be interpreted in the context of local repeat sequence rather than as direct evidence of *TaQT12-D1* regulatory effects. Overall, 42 of the 71 polymorphisms overlapped annotated TEs, including 25 upstream and 17 downstream variants, whereas no intragenic variant overlapped a TE. A complete list of *TaQT12-D1* variants and annotations is provided in Table S6.

**Figure 3:**
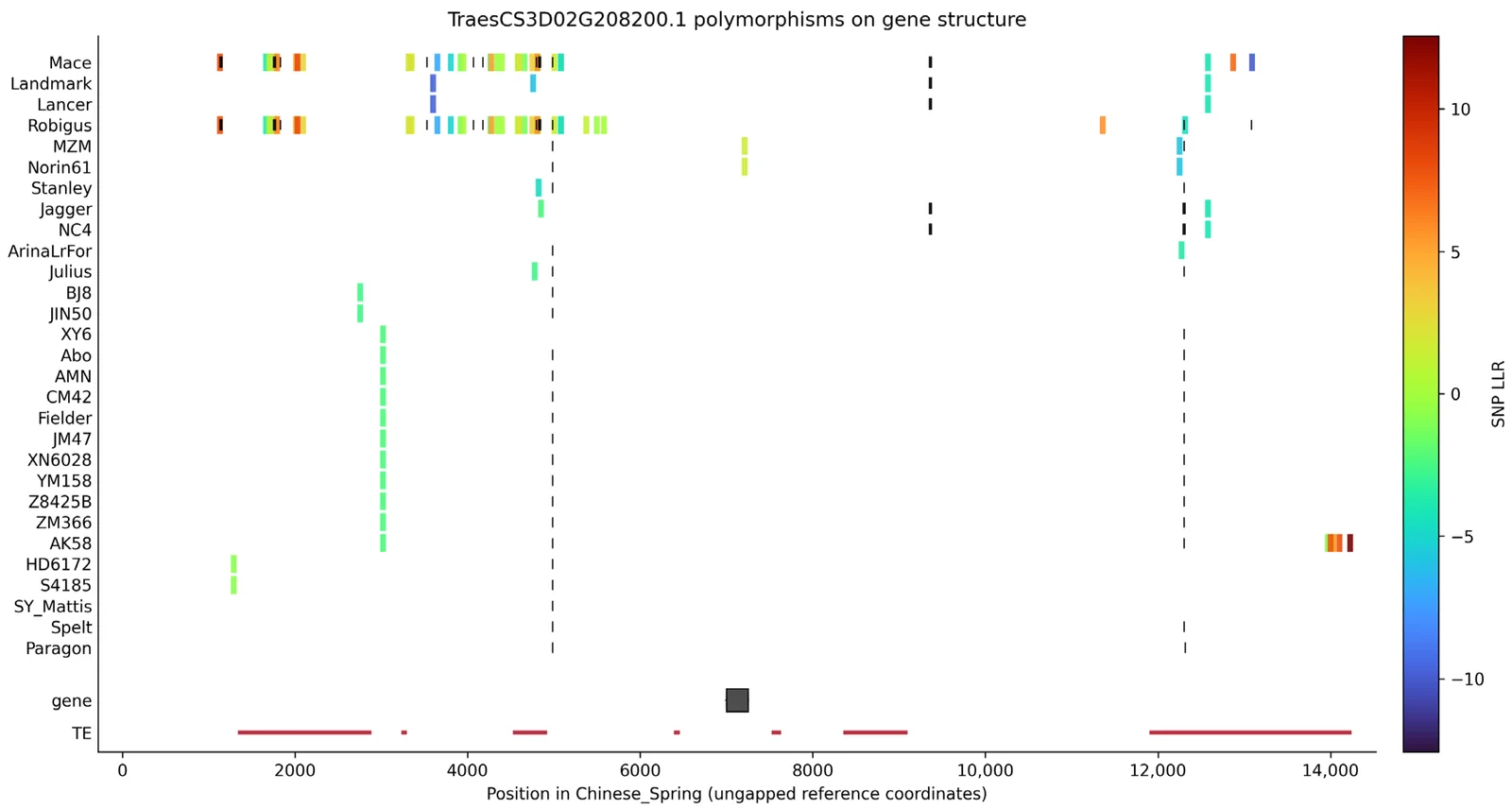
Nucleotide-sequence variation detected around *TaQT12-D1* by comparison of chromosome fragments from hexaploid wheat accessions and zero-shot evaluation of SNVs with AgroNT. Each accession is shown on a separate row. SNVs are colored according to their LLR in the local Chinese Spring reference context (up to ±2500 bp); insertions, deletions, and delins are represented by the symbols described for Figure 2. The gene model, predicted miRNA-binding and cis-element tracks, and annotated TE track are displayed as described for Figure 2.

The only polymorphism within *TaQT12-D1*, chr3D:g.274727990A>G (c.213A>G), was located in the CDS and detected in MZM and Norin 61. Its LLR was +1.51, and the substitution was synonymous and therefore did not alter the amino acid sequence of the protein. No *TaQT12-D1* polymorphism overlapped a predicted miRNA-binding site or detected cis-element site (Figure 3).

Analysis of *TaQT12-B1* variation in the hexaploid wheat pangenome (TraesCS3B02G234900; chr3B:367474744–367480678) separated the analyzed genotypes into two major haplotype groups. The more common group, designated Hap1, comprised 22 of 29 non-reference genomes, including Abo, JIN50, and AK58, and differed substantially from the Chinese Spring reference. The rarer group, Hap2, included Chinese Spring and seven additional genotypes—BJ8, JM47, MZM, S4185, XN6028, Zang1817, and CM42. Within each haplotype group, differences among genotypes were minor. The two groups were distinguished by 108 diagnostic polymorphisms across the analyzed gene-plus-flanking interval. Each diagnostic variant had the alternative state in all 22 Hap1 accessions and the reference state in all eight Hap2 accessions. These variants are explicitly marked in the complete variant table (Table S5). Maximum-likelihood analysis of the pangenome-derived sequences recovered the same two groups (Figure S7b).

In total, 132 unique variants were identified: 112 SNVs, eight insertions, 10 deletions, and two delins. Of these, 71 were located upstream of the transcription start site, 19 within the gene boundaries, and 42 downstream of the transcription termination site. No polymorphisms were detected within the coding sequence: 17 intragenic variants were located in the 5′-UTR intron and two in the 3′-UTR. Eighteen of these 19 variants were characteristic of Hap1. One of the strongest-scoring non-TE intragenic SNVs, chr3B:g.367476844G>A in the 5′-UTR intron (c.-11+2002G>A; LLR = −9.47), was present in all Hap1 accessions and absent from Hap2. This polymorphism did not overlap an annotated TE, predicted miRNA-binding site, or detected cis-element site.

Differences between Hap1 and Hap2 were also pronounced in the proximal upstream region. Within 2 kb upstream of the gene, 14 variants were identified, 12 of which were shared by all Hap1 accessions. Notable variants included chr3B:g.367473637G>C (~1.1 kb upstream; LLR = −4.12), chr3B:g.367473195A>G (~1.55 kb upstream; LLR = −3.09), and chr3B:g.367474353G>A, located only ~0.39 kb upstream of the gene (LLR = −2.57). All three were present in Hap1 and absent from Hap2, and none overlapped an annotated TE, predicted miRNA-binding site, or detected cis-element site. Hap1 was also characterized by two insertions approximately 1.2 and 1.6 kb upstream, for which AgroNT LLR values were not calculated. Only one of the 14 polymorphisms within 2 kb upstream overlapped an annotated TE, indicating that most proximal Hap1/Hap2 promoter-region differences occurred outside annotated transposable elements.

In contrast, TE-associated variation was abundant in the more distal flanking regions. Overall, 53 of the 132 *TaQT12-B1* polymorphisms overlapped TE intervals annotated in Chinese Spring, including 48 upstream and five downstream variants; none of the 19 intragenic variants overlapped these reference TE intervals. Notably, the two SNVs with the most extreme AgroNT scores, chr3B:g.367468588A>G (~6.2 kb upstream; LLR = +13.10) and chr3B:g.367470135A>C (~4.6 kb upstream; LLR = −12.66), were both characteristic of Hap1 and both overlapped annotated TEs. Their extreme LLR values therefore indicate lower or higher compatibility of the alternative allele relative to the reference allele with the local Chinese Spring sequence context, but their occurrence within repeat-associated sequences should be considered when assessing their possible regulatory significance.

**Figure 4:**
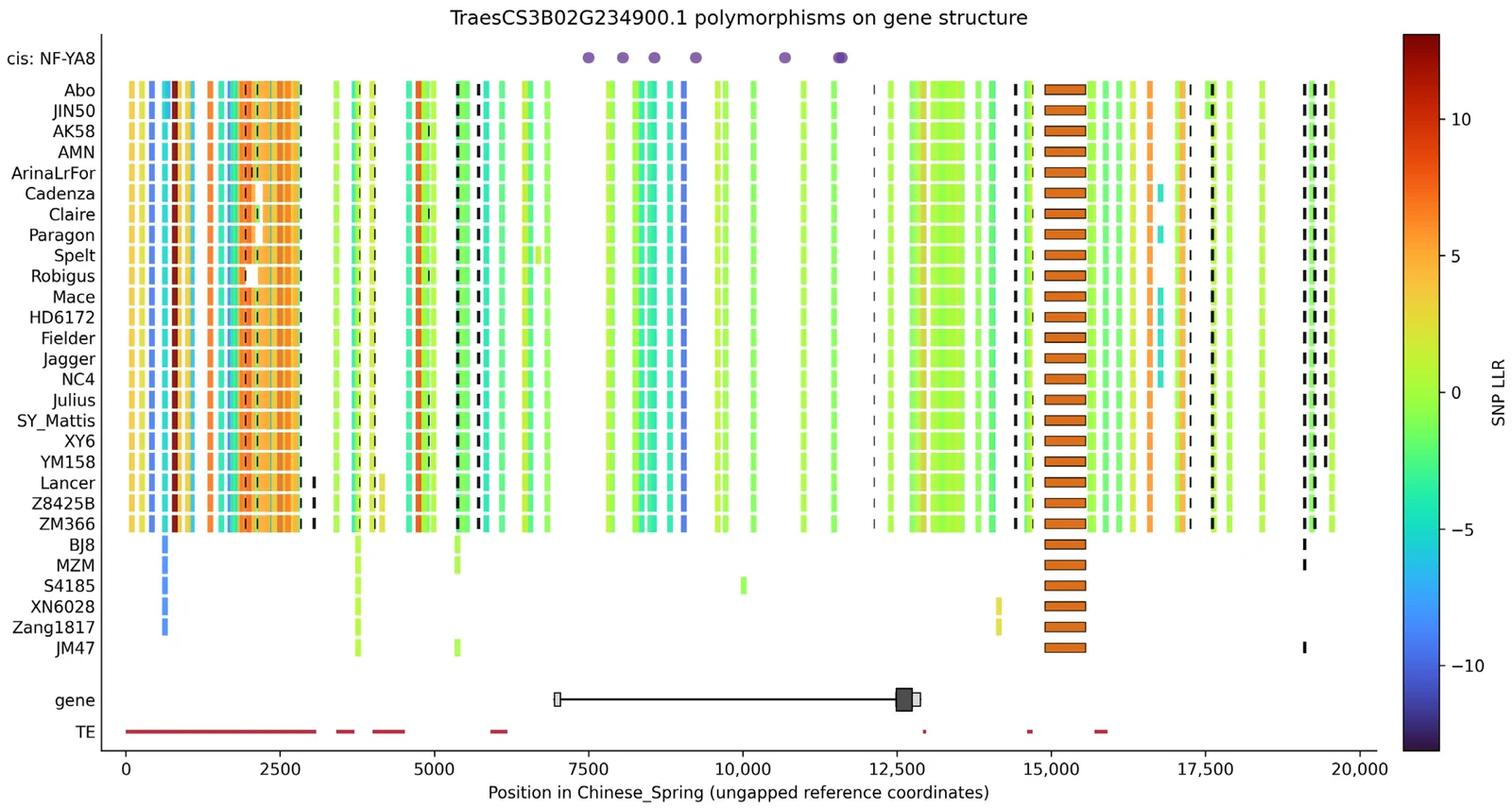
Nucleotide-sequence variation detected around *TaQT12-B1* by comparison of chromosome fragments from hexaploid wheat accessions and zero-shot evaluation of SNVs with AgroNT. The accession-resolved representation clearly distinguishes the major Hap1 group, characterized by numerous differences from Chinese Spring, from the more reference-like Hap2 group. SNVs are shown as LLR-colored vertical bars; insertions, deletions, and delins are represented as described for Figure 2. Predicted miRNA-binding sites and cis-regulatory motifs, when detected, are shown in upper tracks, the *TaQT12-B1* gene model is shown below the accession rows, and annotated transposable elements are displayed as red horizontal segments below the gene model.

None of the 132 *TaQT12-B1* polymorphisms overlapped predicted miRNA-binding sites or detected cis-element sites. Together, these results indicate that the major Hap1/Hap2 differentiation involves extensive non-coding sequence variation, including substantial divergence in distal flanking regions and a distinct set of largely non-TE-associated (based on Chinese Spring reference annotation) polymorphisms in the proximal promoter and 5′-UTR intron, whereas the protein-coding sequence of *TaQT12-B1* remains conserved (Figure 4).

### 2.2. Amplicon Sequencing of QT12 Homeologs

Across the 16 accessions, mean depth within the assessed primer-defined regions ranged from 2313 to 5053× for *TaQT12-A1*, from 1255 to 3073× for *TaQT12-B1*, and from 2209 to 5161× for *TaQT12-D1* (Table S9). The percentages of positions with depth at or above half the corresponding accession-specific mean were 99.964–100% for *TaQT12-A1*, 99.967–100% for *TaQT12-B1*, and 100% for *TaQT12-D1* (Table S10).

Within the assessed primer-defined *TaQT12-A1* regions represented in the mapping reference, no sequence differences from Chinese Spring RefSeq1.0 were detected among the 16 accessions (Figure S2; Table S9).

**Figure 5:**
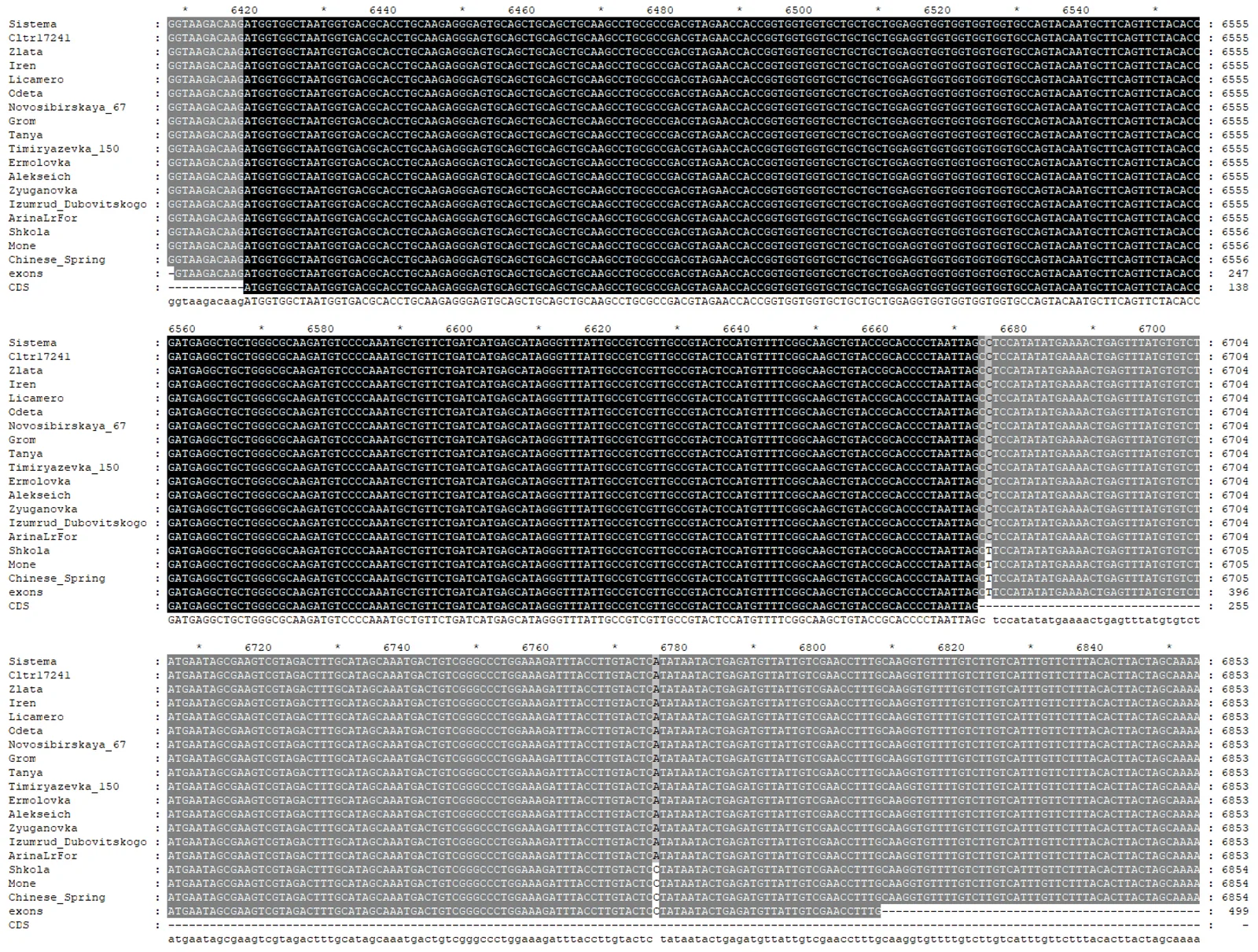
Alignment of *TaQT12-B1* amplicon consensus sequences from the VNIISB collection. Shkola and Mone carry the Chinese Spring-like sequence across the amplified regions, whereas most accessions carry the alternative Hap1-like state; Chinese Spring and ArinaLrFor genome sequences are shown for comparison. The bottom annotation rows indicate exon 2 and the complete Chinese Spring coding sequence (CDS). The two amplicons do not cover the entire long 5′-UTR intron. The numbers above the sequences indicate positions within the alignment, while the asterisks mark every tenth nucleotide.

Sequencing of two *TaQT12-B1* amplicons covering both exons and adjacent non-coding regions revealed two sequence classes among the 16 wheat accessions (Figure S3). Across the sequenced regions, Shkola and Mone carried a Chinese Spring-like sequence; the same local sequence state was also present in the corresponding region of the Norin 61 genome assembly. The remaining accessions carried the alternative Hap1-like sequence: CItr17241 (Chris Mutant), Alekseich, Grom, Ermolovka, Zlata, Zyuganovka, Izumrud Dubovitskogo, Iren, Likamero, Novosibirskaya 67, Odeta, Sistema, Tanya, and Timiryazevka 150. Because the amplicons did not span the entire long 5′-UTR intron, these data do not reconstruct the complete pangenome haplotypes but confirm the major allelic contrast within the experimentally targeted portions of *TaQT12-B1* (Figure 5). Maximum-likelihood analysis of the ONT consensus sequences likewise separated Shkola, Mone, and Chinese Spring from the accessions carrying the Hap1-like sequence (Figure S7a).

Within the assessed *TaQT12-D1* amplicon regions, no differences from Chinese Spring were detected in the cultivars listed in Section 4.1 except for a single-nucleotide insertion, RefSeq1.0.chr3D:g.274727108_274727109insT, in Likamero. The insertion is located 669 bp upstream of the start codon (Figures S4 and S5); no polymorphism was detected in the protein-coding sequence. Its possible regulatory effect was not experimentally assessed and remains unknown.

### 2.3. KASP Marker Development and Haplotype Genotyping

**Figure 6:**
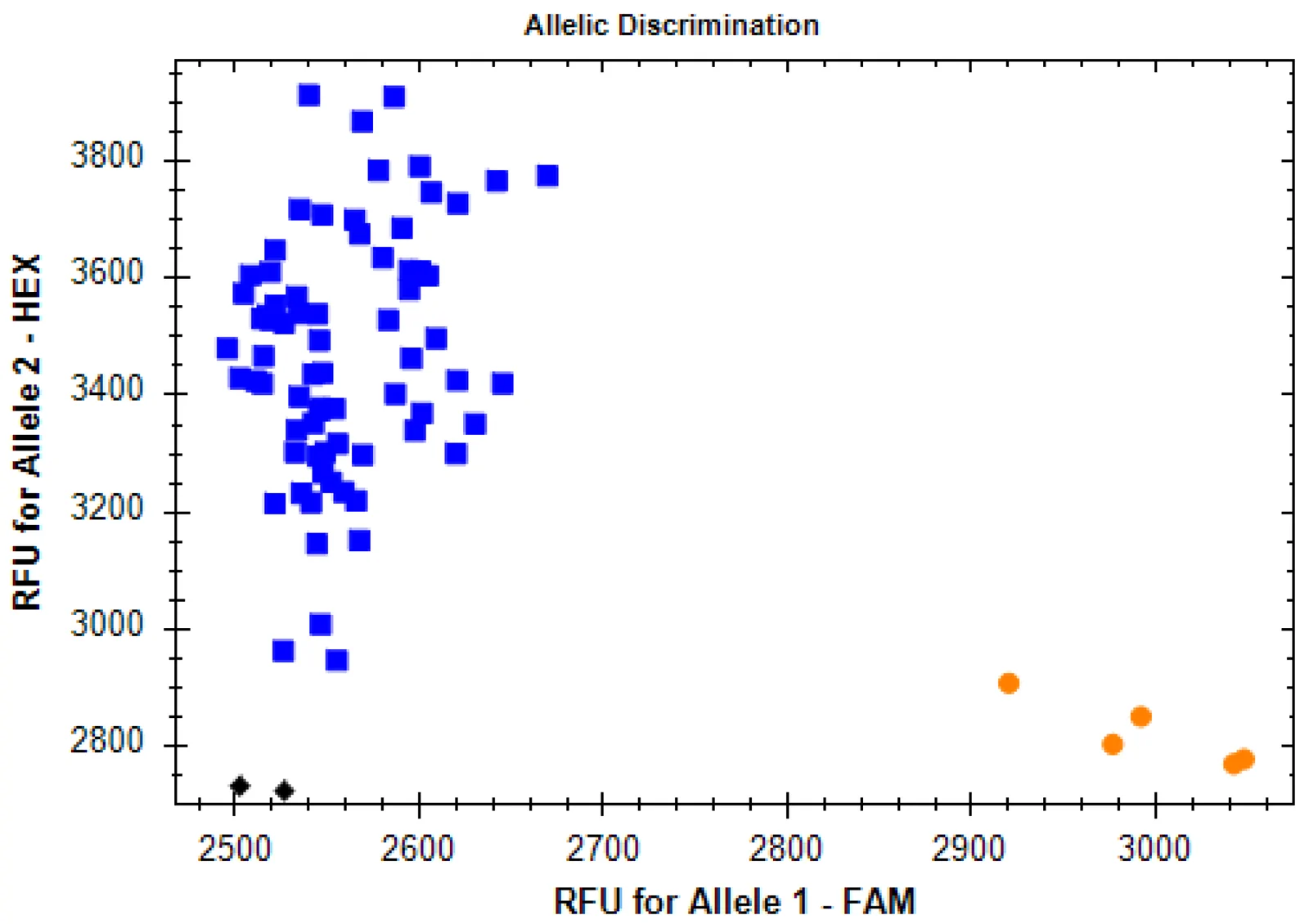
Example of *TaQT12-B1* KASP genotyping results for winter bread wheat accessions from the P.P. Lukyanenko National Grain Center (plate 1). Blue points represent the HEX/G allele corresponding to Hap1, and orange points represent the FAM/C allele corresponding to Hap2.

A KASP marker (Kompetitive Allele-Specific PCR) was developed for the 5′-UTR intron polymorphism c.-10–80C>G (Table 1; Figure 6 and Figure S1). In the haplotypes analyzed here, the C allele detected by FAM corresponded to the Chinese Spring-like Hap2 state, whereas the G allele detected by HEX corresponded to Hap1. Primer specificity relative to the other wheat homeologs is illustrated in Figure S1.

Two hundred winter wheat accessions were genotyped in duplicate with the *TaQT12-B1* KASP marker. Six accessions carried the FAM/C allele corresponding to Hap2—Shkola, Hadm 25612-02, Mone, Evklid, Zhiva, and XIAO YAN 107—whereas all remaining accessions carried the HEX/G allele corresponding to Hap1. KASP allele calls were concordant with ONT sequencing results in all six accessions assessed by both methods (100% concordance; Table S11).

### 2.4. Exploratory Phenotypic Comparison of TaQT12-B1 Haplotypes

**Figure 7:**
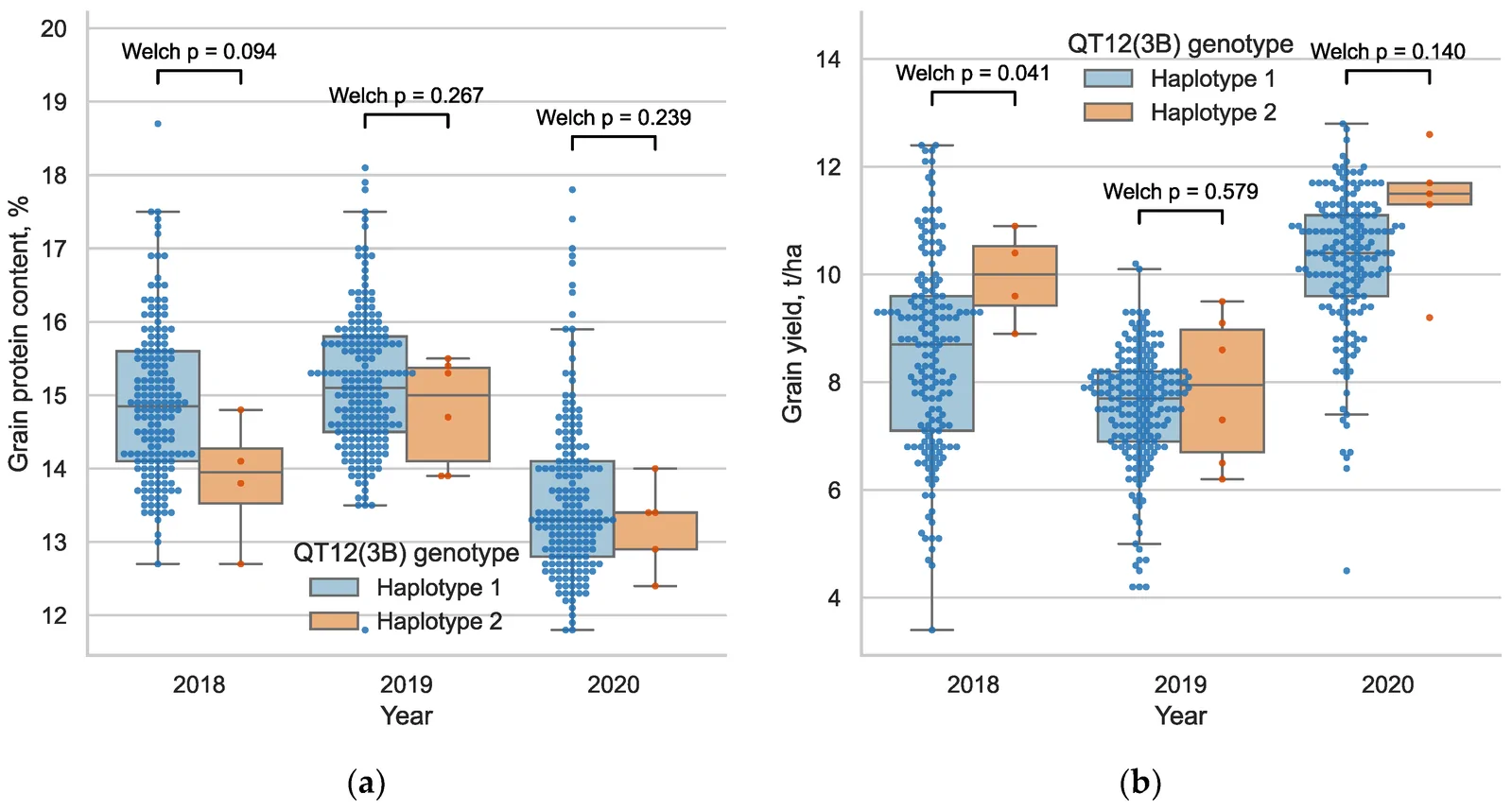
Comparison of winter wheat accessions carrying *TaQT12-B1* Hap1 and Hap2 for grain protein content (**a**) and grain yield (**b**) in 2018–2020. Individual points represent accessions; boxplots summarize the group distributions. Raw two-sided Welch’s t-test *p*-values are shown above the plots. None of the comparisons remained significant after Benjamini–Hochberg FDR adjustment; complete statistics are provided in Table S3.

Exploratory comparisons were performed for six grain-related traits across 2018–2020 (Table S3). Because Hap2 was rare, its effective sample size ranged from four to six accessions depending on trait and year, whereas Hap1 included 168–194 accessions. None of the Hap1–Hap2 contrasts remained statistically significant after Benjamini–Hochberg FDR correction. The lowest raw *p*-value was observed for grain yield in 2018, when the Hap2 mean exceeded the Hap1 mean (9.95 vs. 8.52 t/ha; Welch’s *p* = 0.04; FDR-adjusted *p* = 0.40) (Figure 7).

Several directional patterns were nevertheless consistent across years. Mean grain protein content and thousand-kernel weight were higher in Hap1 in each year, whereas mean grain yield was higher in Hap2 in all three years. Starch content in 2019–2020 and test weight in 2019–2020 also tended to be higher in Hap2, while visual grain quality scores were similar between groups. Figure 7 illustrates the distributions of grain protein content and grain yield. Given the small Hap2 group and the absence of FDR-significant contrasts, these patterns should be regarded as descriptive rather than as evidence of a robust genotype–phenotype association.

### 2.5. Temperature Conditions During the 2018–2020 Field Seasons

**Figure 8:**
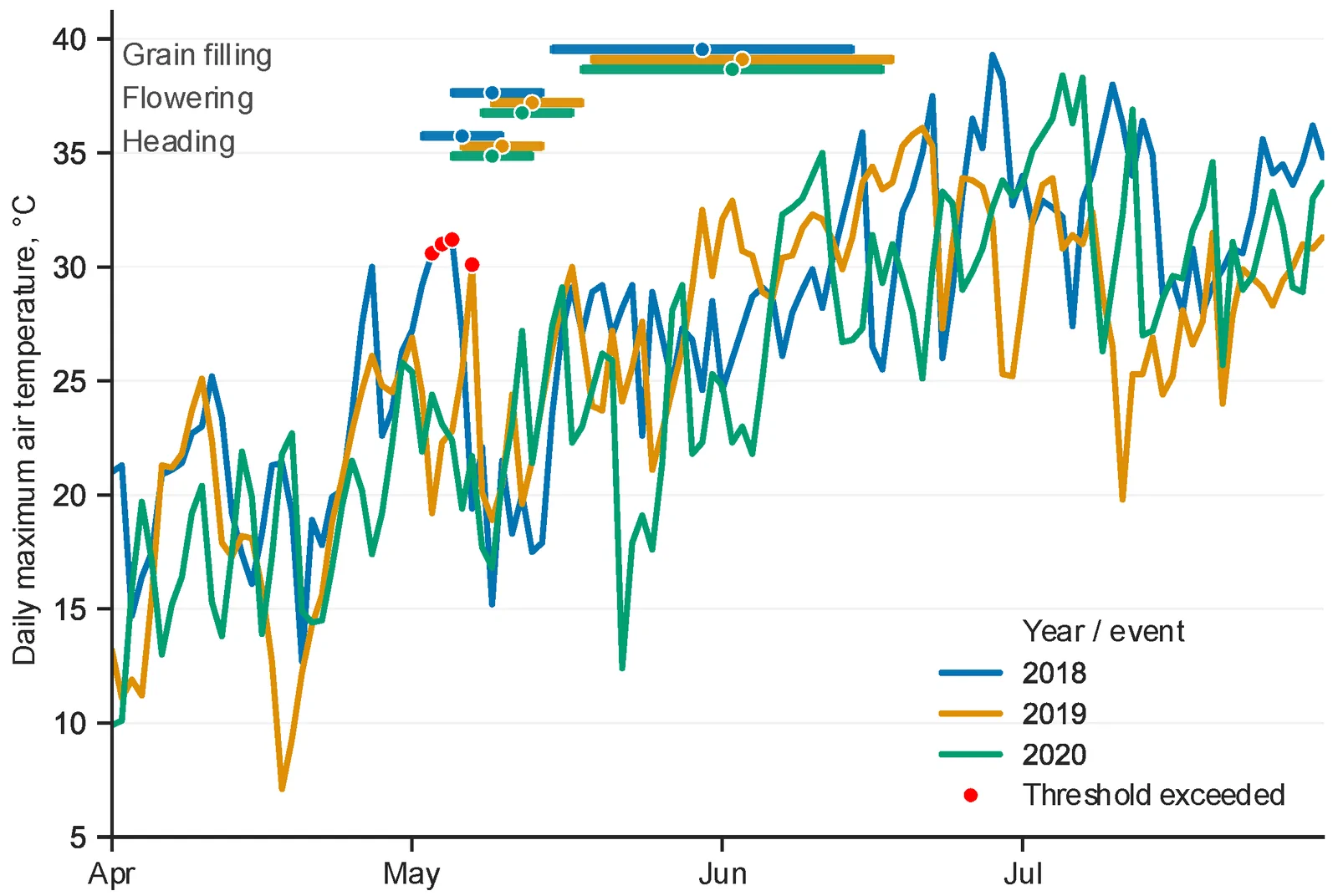
Daily maximum air temperature in spring–summer 2018–2020 at the Krasnodar weather station. Approximate developmental windows for heading, anthesis, and grain filling derived from heading dates, as described in Section 4.7, are shown at the top. Red points mark dates exceeding the phase-specific thresholds used for visualization: 30 °C during heading/anthesis and 35 °C during grain filling.

Thermal conditions during the approximate developmental windows differed substantially among years (Figure 8, Table 2). Using the common 30 °C descriptive threshold in Table 2, 2018 was characterized by earlier threshold exceedances during heading and anthesis, whereas the largest accumulated temperature excess during the grain-filling window occurred in 2019. The 2020 grain-filling period was intermediate between 2018 and 2019 in the number of days above 30 °C and accumulated temperature excess. Figure 8, which uses phase-specific thresholds, likewise shows relatively limited high-temperature exposure around heading/anthesis and greater exposure during grain filling in 2019. These weather records are presented as environmental context for the field seasons and do not constitute a controlled heat-treatment experiment.

## 3. Discussion

The present study was motivated by findings on the rice *QT12* gene, which demonstrated its association with maintenance of grain quality and yield under elevated temperature. *QT12*, which encodes the β subunit of the Sec61 complex, acts as a negative regulator of grain thermotolerance: increased expression at high temperature is accompanied by disruption of storage-compound homeostasis in the endosperm and activation of the endoplasmic reticulum stress response. Natural variants in the *QT12* regulatory region that confer lower expression levels promoted preservation of grain quality and yield under heat. A mechanism of temperature-dependent *QT12* regulation involving the NF-Y transcription-factor complex was also demonstrated [10]. These findings suggest that natural variation in *QT12* homologs may also contribute to heat tolerance in other cereals.

In the present study, the three wheat *QT12* homeologs showed markedly different patterns of natural variation. *TaQT12-A1* and *TaQT12-D1* were highly conserved in their transcribed regions, whereas *TaQT12-B1* separated into two strongly differentiated haplotype groups. The major Hap1/Hap2 contrast did not affect the protein-coding sequence and was concentrated in flanking and untranslated regions, including the proximal upstream region and the long 5′-UTR intron. Most proximal differences occurred outside annotated transposable elements, whereas several of the most extreme AgroNT scores in distal sequences coincided with TEs, emphasizing that LLR reflects local sequence-context compatibility rather than direct regulatory activity. None of the *TaQT12-B1* variants overlapped the NF-Y (CCAAT box) motif or predicted miRNA-binding sites detected in our in-silico analysis. Thus, unlike the rice locus, our analysis did not identify an obvious equivalent of the specific cis-regulatory switch described for rice *QT12*; instead, it defines a broader set of non-coding candidate variants whose effects on expression remain to be tested. In addition, any cis-regulatory effect of *TaQT12-B1* in hexaploid wheat could be buffered by the 3A and 3D homeologs, which may reduce the phenotypic effect of variation at a single homeolog. Both TE-overlap classifications and AgroNT scores are conditional on the Chinese Spring reference. Haplotype-specific TE organization and interactions among linked variants were not assessed. The scores therefore nominate individual substitutions for further study but do not establish functional differentiation between Hap1 and Hap2.

In the pangenome panel, the full-interval Hap2 group showed a marked geographic pattern: Chinese Spring and the seven additional Hap2 accessions were all of Chinese origin. Hap2 was found in modern cultivars as well as in the Tibetan semi-wild wheat Zang1817 [21]. A Chinese Spring-like sequence was also present in the Norin 61 genome assembly across the regions targeted by our amplicons. Hap1 is also widely represented among Chinese cultivars, and Hap2 was detected in accessions of different origins in the 200-accession collection. The geographic pattern here should be interpreted in terms of germplasm history and dissemination rather than as evidence of a specific ecological adaptation [20].

The pronounced divergence between the two *TaQT12-B1* haplotype groups may reflect deep ancestral variation within the wheat B subgenome rather than recent accumulation of mutations. Bread wheat originated through allopolyploidization between tetraploid AABB wheat and *Aegilops tauschii*, and population-genomic studies indicate that the genetic composition of modern bread wheat reflects recurrent hybridization and subsequent gene flow rather than a strictly single-origin history [22,23]. Thus, the marked Hap1/Hap2 divergence at *TaQT12-B1* is consistent with the retention of ancient B-genome variation through the polyploid origin and subsequent evolution, although the present locus alone cannot distinguish recurrent hexaploidization from ancestral polymorphism or later introgression.

The KASP marker developed in this study identifies SNP states associated with the major Hap1/Hap2 groups in the material examined. It showed that Hap1-like haplotypes predominate in the analyzed winter wheat collection, whereas Hap2-like haplotypes were detected in only six of 200 accessions. As a single-SNP assay, it cannot distinguish within-group sequence variants or confirm complete haplotype identity in previously uncharacterized germplasm.

The exploratory phenotype analysis did not identify any Hap1–Hap2 contrast that was statistically significant after FDR correction. Nevertheless, the direction of several mean differences was consistent across years: Hap1 tended to have higher grain protein content and thousand-kernel weight, whereas Hap2 tended to have higher grain yield. However, Hap2 was represented by only six accessions in the genotyped panel and by four to six accessions in individual trait–year comparisons (Table S3). This small sample limits statistical power and the precision of group estimates, making the comparisons potentially sensitive to individual accessions. The observed patterns should therefore be regarded as exploratory hypotheses rather than established phenotypic effects of *TaQT12-B1*. Conversely, the absence of FDR-significant differences should not be interpreted as evidence of phenotypic equivalence between the haplotype groups or of the absence of a yield penalty.

The phenotypic patterns observed here require validation in an independent germplasm panel with substantially greater representation of Hap2. Such validation should account for population structure, genetic background, and developmental timing, and assess the reproducibility of haplotype-associated differences across environments. Any proposed contribution to heat tolerance would additionally require comparisons under matched control and defined heat-stress conditions.

In this study, the largest nominal phenotypic differences were not consistently associated with the year showing the greatest thermal load during the approximate grain-filling window. However, these field trials did not include experimentally imposed heat stress. Seasonal weather records provide environmental context but do not allow temperature effects to be separated from other differences among years. Consequently, the present phenotypic comparisons do not establish differences in heat tolerance between Hap1 and Hap2.

Any phenotypic differences between the haplotype groups may also reflect genetic factors other than *TaQT12-B1* itself. The two haplotypes differ across an extended sequence interval, so the KASP marker may tag linked variation in neighboring loci as well as variants within *TaQT12-B1*. Grain yield, kernel weight, and grain composition are also influenced by developmental timing and broader cultivar background. In addition, the tendency toward higher grain protein content in Hap1 together with somewhat lower yield could partly reflect the well-known negative relationship between wheat yield and grain protein concentration rather than a specific temperature-response mechanism.

Genetic variation on chromosome 3B has previously been linked to heat-response traits in wheat. For example, a QTL affecting stability of grain weight after a brief heat treatment early in grain filling was mapped to the short arm of chromosome 3B [24]. This QTL does not provide positional evidence for a role of *TaQT12-B1* in heat tolerance.

Further work should focus on direct functional characterization of the locus. Particularly informative would be the comparison of homeolog-specific *QT12* expression in Hap1 and Hap2 backgrounds under control and elevated-temperature conditions, especially during grain development. Broader screening of diverse germplasm and genetic materials that separate *TaQT12-B1* from linked background variation would help determine whether the identified non-coding haplotypes have reproducible phenotypic effects.

## 4. Materials and Methods

### 4.1. Plant Material

The wheat accessions from the collection of All-Russia Research Institute of Agricultural Biotechnology (VNIISB) used for sequencing the *QT12* homeologs are listed in Table 3.

To assess *TaQT12-B1* haplotype frequency and explore associations with agronomically important traits, we used 200 winter bread wheat accessions provided by the P.P. Lukyanenko National Grain Center together with annual phenotype data collected in 2018–2020. Field-trial design and phenotyping procedures for this collection were described previously [25,26]. The field trials were conducted under naturally varying seasonal conditions, without experimentally imposed heat-stress treatments. The present analysis used accession-level annual trait values. Available traits included heading date, plant height (cm), grain yield (t/ha), thousand-kernel weight (g), grain protein content (%), grain gluten content (%), grain starch content (%), test weight (g/L), and an overall visual grain quality score (1 indicates poor grain and 9 indicates excellent grain). The underlying accession-level phenotype dataset used in this study is provided in Table S8.

### 4.2. DNA Extraction

Fresh seedlings obtained by germinating grains on moist filter paper were homogenized using a TissueLyser II (QIAGEN GmbH, Hilden, Germany). Total DNA was isolated with an Auto-Pure 96 automated nucleic acid extraction and purification system (Hangzhou Allsheng Instruments Co., Ltd., Hangzhou, China) using the MagnoPrime^®^ GMO DNA extraction kit (NextBio LLC, Moscow, Russia), according to the manufacturer’s protocol adapted for automated extraction.

### 4.3. Selection of Wheat QT12 Homologs and PCR Primer Design

Candidate wheat homologs were evaluated using Ensembl Plants Compara orthology output for rice *QT12* (LOC_Os12g07490; Os12g0173366 in the Ensembl gene nomenclature). Six bread wheat co-orthologs were identified, forming homeologous triads on chromosome groups 3 and 5: *TraesCS3A02G207800*, *TraesCS3B02G234900*, *TraesCS3D02G208200*, *TraesCS5A02G015200*, *TraesCS5B02G013400*, and *TraesCS5D02G021100*. Publicly available RNA-seq expression data for these six genes were obtained from the Wheat Expression Browser/expVIP and compared across the aggregated tissue and stress categories supplied by the database (Figure S6; [16,17]). Because the chromosome group 3 triad generally showed higher expression than the group 5 triad, and *TraesCS3B02G234900* showed the highest mean expression in most categories, the chromosome group 3 triad was selected for detailed sequence analysis.

Because all coordinate-based analyses were performed against the Chinese Spring RefSeq1.0 assembly, RefSeq1.0 gene identifiers are used throughout the “Section 2 and Section 4: TraesCS3A02G207800 (*TaQT12-A1*), TraesCS3B02G234900 (*TaQT12-B1*), and TraesCS3D02G208200 (*TaQT12-D1*). The corresponding RefSeq2.1 identifiers are TraesCS3A03G0538900, TraesCS3B03G0601500, and TraesCS3D03G0491700, respectively.

**Figure 9:**
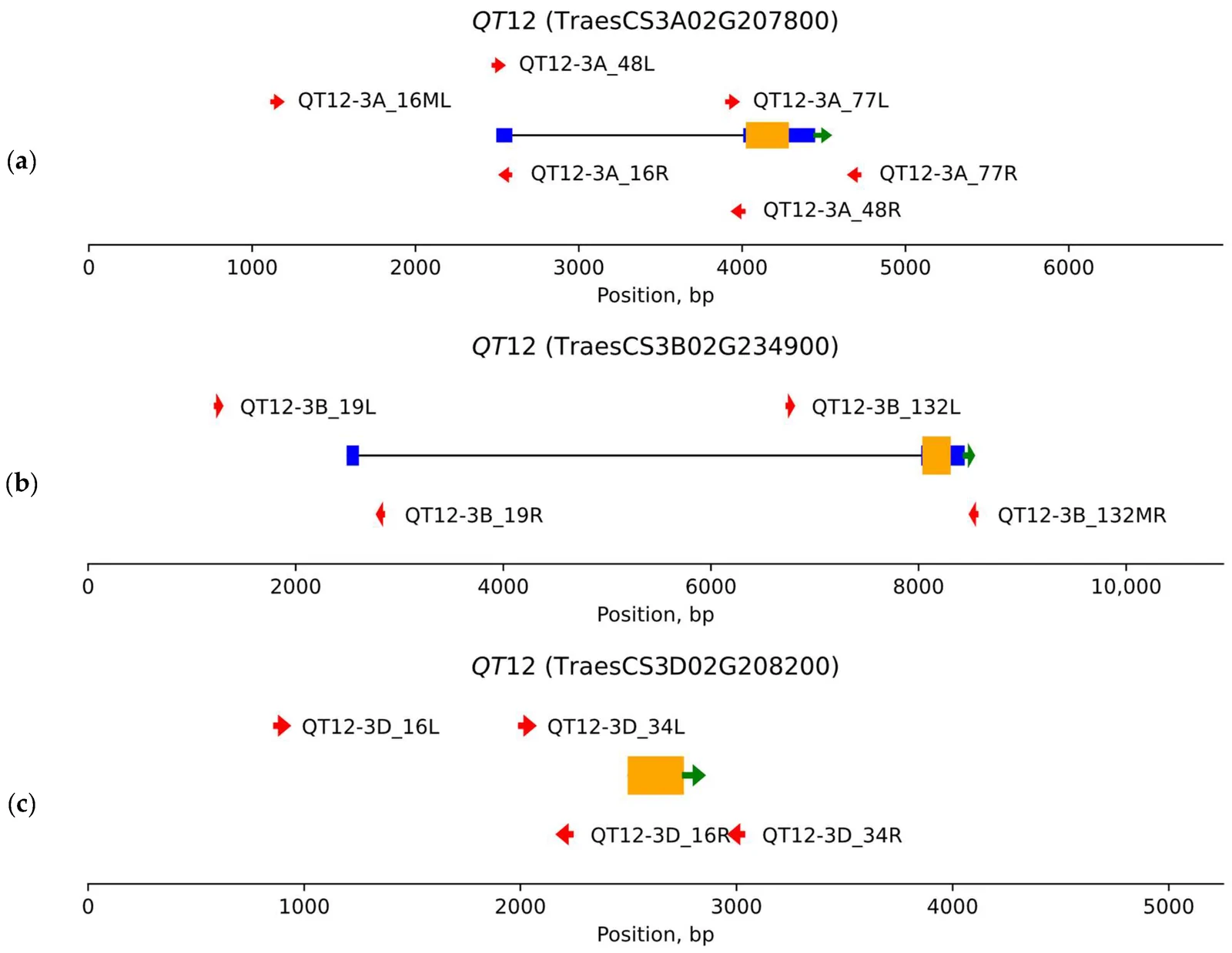
Relative positions of primers and genes in chromosome-region diagrams containing wheat *QT12* homeologs. Genes are represented as sets of rectangles denoting exons connected by lines denoting introns. Orange rectangles indicate protein-coding DNA regions, whereas blue rectangles indicate regions corresponding to untranslated regions of mature mRNA. Primers are shown as red arrows. Chromosome 3A region containing TraesCS3A02G207800 (**a**); chromosome 3B region containing TraesCS3B02G234900 (**b**); chromosome 3D region containing TraesCS3D02G208200 (**c**).

Primers for gene fragment amplification were designed using a software pipeline based on the Primer3 (v.2.4.0) primer-design tool, the RefSeq1.0 wheat genome assembly, and its annotation. The pipeline performs the following steps: (1) identification of homeologous genes and their chromosomal coordinates from gene identifiers in the annotation files; (2) extraction of chromosome fragments containing the genes together with flanking sequences of a specified length; (3) masking of repetitive sequences, including mobile genetic elements, based on genome annotation; (4) identification and masking of short tandem repeats to exclude repetitive regions from primer design; (5) design of primer pairs with Primer3 to generate PCR products of a specified maximum length for successive overlapping fragments of the target chromosome sampled at defined intervals; and (6) selection of an optimal set of primer pairs covering the gene and its flanking sequences with the minimum number of overlapping PCR products. Primer specificity for the target sequence was checked during Primer3 design. Homeologous genes from the other two wheat subgenomes were used as off-target sequences. The primers selected in this way and actually used for PCR amplification of wheat *QT12* homeolog fragments are listed in Table S2, and their relative positions on the gene diagrams are shown in Figure 9. To visualize gene structure and primer positions relative to gene features, the chromosome, target gene, exon, and protein-coding sequences were aligned using MUSCLE [27] and used to make gene diagrams with a custom function in Python 3.10.21.

Primers for the KASP marker distinguishing *QT12* haplotypes on chromosome 3B were designed using Primer-BLAST (NCBI), and their specificity was assessed by visual inspection of an alignment of the gene haplotypes and their homeologs in GeneDoc 2.7 [28].

### 4.4. PCR Conditions and Product Detection

#### 4.4.1. Amplicon Preparation for Sequencing

The PCR mixture for one sample contained 6.25 μL ddH2O, 2 μL dNTPs (2.5 mM), 12.5 μL 2× Encyclo GC buffer (Evrogen, Russia), 0.75 μL Encyclo polymerase (Evrogen, Russia), 1.5 μL DNA template at 100 ng/μL, and 1 μL of each primer (10 μM), resulting in a total volume of 25 μL. PCR conditions were as follows: initial denaturation, one cycle at 98 °C for 5 min; touchdown, five cycles at 97 °C for 30 s, 63 °C for 30 s with a 1 °C decrease in annealing temperature per cycle, and 72 °C for 5 min; 35 cycles at 97 °C for 30 s, 58 °C for 30 s, and 72 °C for 5 min; and final extension, one cycle at 72 °C for 10 min. PCR was performed using a T100 Thermal Cycler (Bio-Rad Laboratories, Inc., Hercules, CA, USA).

PCR products were evaluated by electrophoresis in a 1.5% agarose gel in TBE buffer and stained with ethidium bromide. A 1 kb DNA Ladder (Evrogen CJSC, Moscow, Russia) was used as a molecular size marker. Results were visualized using a BIO-RAD Gel Doc XR+ transilluminator and Image Lab v6.0.1, build 34 software. The resulting amplicons were used for gene sequencing.

#### 4.4.2. KASP Marker

A bulk reaction premix was prepared by combining KASP 2× Mix KBS-1050-102 (containing FAM, HEX, and ROX dyes; LGC Biosearch Technologies/LGC Genomics Ltd., Hoddesdon, Hertfordshire, UK) and primer mix at a volume ratio of 5:0.14. Each PCR tube contained 5 μL of this premix and 5 μL of DNA template at 50 ng/μL, which gave a total reaction volume of 10 μL. Amplification conditions were as follows: one cycle at 94 °C for 15 min; nine cycles at 94 °C for 20 s and 60 °C for 1 min, with a 0.6 °C decrease per cycle; 35 cycles at 94 °C for 20 s and 55 °C for 1 min; and 37 °C for 1 min, during which fluorescence was read. KASP assays were performed using a BIO-RAD CFX96 real-time PCR system and CFX Manager v3.1 software.

### 4.5. Oxford Nanopore Sequencing and Sequence Processing

PCR products belonging to the same wheat accession were pooled. PCR products were purified from the reaction mixture using HighPrep PCR beads (MagBio Genomics, Inc., Gaithersburg, MD, USA). DNA concentration was measured on a Qubit 4.0 fluorometer (Thermo Fisher Scientific, Waltham, MA, USA) using the dsDNA Quantification Broad Range Kit (BioDynami Corporation, Huntsville, AL, USA). PCR products from different wheat accessions were barcoded using the Native Barcoding Kit 24 V14 (SQK-NBD114.24, Oxford Nanopore Technologies plc, Oxford, UK).

Samples were sequenced on a FLO-MIN114 flow cell (Oxford Nanopore Technologies, UK) using a GridION sequencer (Oxford Nanopore Technologies, Oxford, UK) controlled by MinKNOW v.24.11.16. Basecalling, demultiplexing, and adapter trimming were performed with dorado v.1.4.0 (https://github.com/nanoporetech/dorado; accessed on 14 May 2026) using the dna_r10.4.1_e8.2_400bps_sup@v5.2.0 model.

Sequencing data were processed by mapping reads to a custom reference containing chromosome fragments from Chinese Spring RefSeq1.0 encompassing *TaQT12-A1*, *TaQT12-B1*, and *TaQT12-D1*, identifying sequence variants (mutations), and incorporating these variants into consensus sequences. Before mapping, the reference sequences were indexed using GATK v4.6.2.0 and samtools 1.21.

Reads were aligned to the reference using minimap2 2.26-r1175 with the ‘map-ont’ preset appropriate for the sequencing technology used. Alignments were converted to BAM format with ‘samtools view’, retaining reads with mapping quality of at least 10, and sorted with ‘samtools sort’. Insertions and deletions were left-aligned using GATK ‘LeftAlignIndels’.

Sequence variants were called against the mapping reference using FreeBayes v1.3.8. Variants with QUAL < 200 were excluded using bcftools 1.19. Retained variants were normalized against the reference using bcftools norm, and the resulting VCF files were indexed using tabix 1.21.

Read alignments were visually inspected in IGV 2.16.1 [29] to assess potentially nonspecific read contributions and ambiguous consensus positions. Ambiguous consensus positions were reviewed manually against the local read alignments.

Consensus sequences were generated by incorporating retained variants into the mapping reference using bcftools consensus. Per-position read depth was calculated in reference coordinates using samtools depth with the -aa option. The original consensus-processing workflow used an absolute depth threshold of 20× to flag low-depth sequence with lowercase letters; these positions were excluded when interpreting sequence conservation and the absence of variants.

For coverage assessment, target intervals were defined using primer positions and expected amplicon spans (Table S2 and Figure 9). Primer-binding positions were included, overlapping amplicons were counted once, and amplicon portions outside the reference fragments were not assessed. Mean, minimum, and maximum depth were calculated across all positions within these intervals, without a 20× depth cutoff (Table S9). For each accession and homeolog, we additionally calculated the percentage of target positions with depth below half the corresponding mean depth to show depth uniformity (Table S10). Consensus sequences were aligned using MUSCLE 5.1 [27].

### 4.6. Pangenome Sequence Retrieval and Comparative Analysis

Complete wheat genome assemblies were downloaded from publicly available sources (Table S1) [19,20,30,31,32,33,34,35,36]. For each homeolog, the target locus was identified with blastn (Nucleotide-Nucleotide BLAST 2.15.0+) using the corresponding Chinese Spring RefSeq1.0 sequence as the query. The resulting coordinates were used to extract the gene together with 7000 bp of upstream and 7000 bp of downstream flanking sequence. Assemblies were retained for a given homeolog only when the target locus and sufficient contiguous flanking sequence could be recovered unambiguously; consequently, the number of usable assemblies could differ among loci. Extracted sequences were aligned using MUSCLE 5.1.

*TaQT12-B1* sequences were assigned to Hap1 or Hap2 by visual inspection of the multiple-sequence alignment and the distribution of variants relative to Chinese Spring (Figure 4). Diagnostic polymorphisms were defined as variants for which the alternative state was present in all 22 Hap1 accessions and the reference state was present in all eight Hap2 accessions, including Chinese Spring. These diagnostic polymorphisms were identified using the pangenome sequences only and are explicitly marked in Table S5.

The grouping was additionally evaluated by maximum-likelihood phylogenetic analysis in MEGA11 [37] using the Tamura–Nei substitution model and 500 bootstrap replicates. Separate trees were reconstructed for the pangenome-derived sequences and the ONT amplicon consensus sequences, with Chinese Spring included in both datasets. Positions containing gaps or missing data were excluded using the complete-deletion option (Figure S7).

Transposable-element (TE), miRNA-binding-site, and cis-regulatory motif annotations were additionally integrated into the polymorphism analysis. TE coordinates and mature wheat miRNA sequences were obtained from the Chinese Spring IWGSC RefSeq v1.0 annotation [38]. TE-overlap assignments were based exclusively on the Chinese Spring annotation, with variant positions related to the reference through the multiple-sequence alignment. No accession-specific TE reannotation was performed. Putative miRNA-binding sites were searched separately in each accession represented in the multiple-sequence alignment. For this purpose, the mature mRNA sequence of each accession was reconstructed by concatenating the exon regions defined by the reference transcript annotation, and the reverse-complement sequence of each mature miRNA was searched against the resulting spliced transcript, allowing up to two nucleotide mismatches and no insertions or deletions. Cis-regulatory motifs were searched in the complete gene-region sequence of each aligned accession, including both exonic and intronic regions. One predefined motif was considered: the CCAAT box, corresponding to a potential NF-YA8-binding site [10]. Motif searches were performed in both sequence orientations. The coordinates of TE intervals, predicted miRNA-binding sites, and detected cis-regulatory motifs were finally compared with polymorphism positions and incorporated into the annotation and visualization shown in Figure 2, Figure 3 and Figure 4. Complete variant tables generated from these comparisons are provided in Tables S4–S6.

### 4.7. Weather Data and Approximate Developmental Windows

Daily maximum air temperature (Tmax, °C) was obtained from the archive of the ‘Weather and Climate’ information portal (www.pogodaiklimat.ru) for the Krasnodar weather station (WMO 34927; latitude 45.0407, longitude 39.1531, elevation 29 m) for April–July 2018–2020. Data were visualized in Python 3.10.21 using pandas 2.3.3, Seaborn 0.13.2, and Matplotlib 3.10.9. For Figure 8, phase-specific upper-temperature thresholds of 30 °C for heading/anthesis and 35 °C for grain filling were used based on Porter and Gawith [3] and Groli et al. [39]. Because daily observations of anthesis and grain-filling onset were not available for each accession, developmental windows were approximated from the recorded heading dates. For each year, the heading window was defined as the mean heading date across accessions ± one standard deviation; the anthesis window of the same duration was shifted three days later, and grain filling was represented by the subsequent 30-day period. These windows were used only to describe seasonal thermal conditions and not as genotype-specific heat treatments. For the descriptive summary in Table 2, a common threshold of 30 °C was used across all three developmental periods to facilitate comparison. Accumulated temperature excess was calculated as Σ(Tmax − 30 °C) for days on which Tmax exceeded 30 °C. The underlying daily temperature series is provided in Table S7.

### 4.8. Statistical Analysis of Phenotypic Traits

Phenotypic traits of accessions carrying *TaQT12-B1* Hap1 and Hap2 were analyzed separately for each year (2018–2020). Missing observations were excluded, and the resulting sample size was reported for each trait-year comparison (Table S3). Differences between Hap1 and Hap2 were assessed using two-sided Welch’s t-tests for independent samples, which do not assume equal variances. To account for multiple comparisons, the Benjamini–Hochberg false discovery rate (FDR) procedure was applied jointly to the *p*-values from all 17 Hap1–Hap2 trait–year comparisons reported in Table S3, treating them as a single family of tests across 2018–2020. FDR-adjusted *p* < 0.05 was considered statistically significant. Because Hap2 was represented by only a small number of accessions, these comparisons were treated as exploratory. Data were visualized in Python using Seaborn and Matplotlib, with boxplots overlaid with swarmplots to display individual observations.

### 4.9. AgroNT Scoring of Single-Nucleotide Variants

To characterize the sequence-context compatibility of single-nucleotide variants (SNVs), we used the pretrained genomic language model AgroNT (InstaDeepAI/agro-nucleotide-transformer-1b) in a zero-shot masked-language-modeling setting without additional training. For each SNV, a reference-sequence context of up to 2500 bp on each side of the polymorphism was extracted; where the full context was unavailable, the available sequence was used. The token containing the SNV was masked, and the model was used to obtain scores for the reference and alternative states. The substitution score was expressed as a log-likelihood ratio, LLR = log P(ALT | context) − log P(REF | context), equivalent to the difference between the corresponding unnormalized model output scores (logits). Negative LLR values therefore indicate greater compatibility of the reference allele with the local sequence context, whereas positive values indicate greater compatibility of the alternative allele. Each SNV was evaluated individually in the Chinese Spring reference sequence context, without introducing the other linked variants present in the alternative haplotype. LLR scores were used to assess local sequence-context compatibility, rather than as direct predictions of regulatory effects or of the combined effects of multiple linked variants within complete alternative haplotypes. Insertions, deletions, and extended replacement variants (delins) were not scored.

Custom code used for data collection, primer design, sequence processing and annotation, calculations and visualizations in this research is available at [40]. GPT-5.6 Sol large language model was used to assist with Python code development.

## 5. Conclusions

In conclusion, bread wheat *TaQT12-B1* contains two strongly differentiated haplotype groups whose major differences are non-coding, while the protein-coding sequence remains conserved. The KASP marker developed here enables rapid discrimination of these haplotypes and showed that Hap2 is rare in the analyzed winter wheat collection. Exploratory phenotypic comparisons and seasonal temperature data did not provide robust evidence that either haplotype confers heat tolerance. The principal contribution of this study is therefore the definition of *QT12* homeolog diversity and the establishment of sequence and marker resources for direct functional testing of this candidate locus.

## Figures and Tables

**Table 1:** Primer sequences for KASP genotyping of the *TaQT12-B1* 5′-UTR intron polymorphism c.-10–80C>G.

Primer	Sequence (5′→3′) *
TaQT12-B1-FAM	GAAGGTGACCAAGTTCATGCTGATGCATGCCGTTTGATCATTTT**C**
TaQT12-B1-HEX	GAAGGTCGGAGTCAACGGATTGATGCATGCCGTTTGATCATTTT**G**
TaQT12-B1-com	CTACCTACATGGAAGTGCAAGGT

* Adapter sequences required for interaction with the FRET-cassette oligonucleotides are underlined, and the 3′-terminal nucleotide detected by the marker is shown in bold. Product length, including the adapters, is 124 bp.

**Table 2:** Descriptive indices of thermal conditions during approximate heading, anthesis, and grain-filling windows in 2018–2020. A common 30 °C threshold was used for the exceedance-based indices to facilitate comparison among developmental periods.

	Mean Daily Maximum Temperature During the Period, °C			Number of Days with Maximum Temperature Above 30 °C			Accumulated Temperature Excess Above 30 °C, °C·Day		
Year	2018	2019	2020	2018	2019	2020	2018	2019	2020
Heading	25.3	22.7	21.1	3	1	0	2.8	0.1	0.0
Flowering	21.0	23.8	23.0	1	0	0	1.2	0.0	0.0
Grain filling	27.9	29.2	25.8	3	16	6	6.3	33.4	18.3

**Table 3:** Bread wheat (*Triticum aestivum* L.) accessions used for sequencing the *QT12* homeologs.

Registry Code *	Accession Number **	Cultivar/Line	Growth Habit	Origin
9360103	K-65223	Grom	winter	P.P. Lukyanenko National Grain Center (Krasnodar, Russia)
9811733	K-64589	Tanya	winter	
8154722	—	Shkola	winter	
8154715	—	Mone	winter	
8458118	—	Timiryazevka 150	winter	
8556858	K-66806	Alekseich	winter	
7853071	—	Ermolovka	winter	Shchelkovo Agrokhim JSC (Shchelkovo, Russia)
—	—	Zyuganovka	winter	
7953294	—	Izumrud Dubovitskogo	winter	
7853690	—	Sistema	winter	
—	CItr 17241	Chris Mutant	spring	Minnesota Agricultural Experiment Station (St. Paul, MN, USA)
9464217	K-66414	Zlata	spring	Federal Research Center Nemchinovka (Nemchinovka, Russia)
9601678	K-62633	Iren	spring	Ural Federal Agrarian Research Center, Ural Branch of the Russian Academy of Sciences (Yekaterinburg, Russia)
8558204	K-67099	Likamero	spring	SECOBRA RECHERCHES S.A.S. (Maule, France)
8355952	K-66394	Odeta	spring	SELGEN A.S. (Prague, Czech Republic)
—	K-48601	Novosibirskaya 67	spring	Institute of Cytology and Genetics, Siberian Branch of the Russian Academy of Sciences (Novosibirsk, Russia)

* State Register of Varieties and Hybrids of Agricultural Plants Approved for Use in the Russian Federation as of 11 June 2026. A dash indicates that no registry code or genebank accession number was identified, as applicable. ** Accession number prefixes: K, collection of the N.I. Vavilov All-Russian Institute of Plant Genetic Resources (VIR), Russia; CItr, Cereal Investigation number for wheat (Triticum) in the USDA National Plant Germplasm System, USA, as recorded in GRIN-Global.

## Data Availability

The original contributions presented in this study are included in the article/Supplementary Materials. Further inquiries can be directed to the corresponding author.

## Supplementary Materials

The following supporting information can be downloaded at: https://www.mdpi.com/article/10.3390/plants15213345/s1, Table S1, The list of complete wheat genome assemblies used to study *QT12* diversity; Table S2, Primer pairs used for amplicon sequencing; Figures S1–S5, KASP-primer and ONT-sequencing alignments; Table S3, Haplotype-group phenotype statistics; Figure S6, Expression profiles of the six wheat co-orthologs of rice *QT12*; Figure S7, Maximum-likelihood phylogenetic trees of *TaQT12-B1*; Tables S4–S6, Complete pangenome variant datasets for *TaQT12-A1*, *TaQT12-B1*, and *TaQT12-D1*; Table S7, Daily maximum air-temperature data for Krasnodar; Table S8, Accession-level phenotype data; Table S9, Mean and minimum–maximum ONT read depth across primer-defined target regions; Table S10, Percentages of target positions below half the accession-specific mean depth; Table S11, Concordance between ONT sequencing and KASP genotyping. Pangenome-derived and ONT consensus sequences are provided as separate FASTA files in the supplementary sequence archive.

## Abbreviations

The following abbreviations are used in this manuscript:

AI	artificial intelligence
ALT	alternative allele/state
BAM	Binary Alignment/Map
BLAST	Basic Local Alignment Search Tool
CDS	coding sequence
ddH_2_O	double-distilled water
DNA	deoxyribonucleic acid
dNTP(s)	deoxyribonucleoside triphosphate(s)
ER	endoplasmic reticulum
FAM	6-carboxyfluorescein
FDR	false discovery rate
FRET	Förster resonance energy transfer
GATK	Genome Analysis Toolkit
HEX	hexachlorofluorescein
HSE	heat-shock element
IGV	Integrative Genomics Viewer
IWGSC	International Wheat Genome Sequencing Consortium
KASP	Kompetitive Allele-Specific PCR
LLR	log-likelihood ratio
miRNA	microRNA
MUSCLE	Multiple Sequence Comparison by Log-Expectation
NCBI	National Center for Biotechnology Information
NF-Y	Nuclear Factor Y
NF-YA8	Nuclear Factor Y subunit A8
ONT	Oxford Nanopore Technologies
PCR	polymerase chain reaction
PDB	Protein Data Bank
QTL	quantitative trait locus
REF	reference allele/state
RefSeq	Reference Sequence
RNA	ribonucleic acid
RNA-seq	RNA sequencing
ROX	carboxy-X-rhodamine
SNV	single-nucleotide variant
TBE	Tris-borate-EDTA
TE	transposable element
Tmax	daily maximum air temperature
TPM	transcripts per million
UTR	untranslated region
VCF	Variant call format
VNIISB	All-Russia Research Institute of Agricultural Biotechnology
WMO	World Meteorological Organization
