# Supplementary material for: Natural Variation in Wheat Homologs of the Rice Thermotolerance Gene QT12 Reveals Two Divergent Haplotypes of TaQT12-B1
Source: Plants (Basel). 2026 Oct 28;15(21):3345. doi: 10.3390/plants15213345 (PMC13766174; doi:10.3390/plants15213345)
Supplement: Supplementary file 1 [file plants-15-03345-s001.zip › Supplementary_materials.pdf]

Supplementary Materials

Table S1. The list of complete wheat genome assemblies used to study *QT12* diversity.

No.	Accession	Accession number*	Species	Geographic origin**	Growth habit**	Assembly level	Reference
1	Abo	PI 351699	*T. aestivum*	Italy	spring	chromosome	[1]
2	AK58	—	*T. aestivum*	China	winter	chromosome	[2]
3	AMN	—	*T. aestivum*	China	winter	chromosome	[1]
4	ArinaLrFor	PANG0001	*T. aestivum*	Switzerland	winter	chromosome	[3]
5	BJ8	ZM008963	*T. aestivum*	China	winter	chromosome	[1]
6	Cadenza	PANG0002	*T. aestivum*	United Kingdom	spring	scaffold	[3]
7	CDC Landmark	PANG0003	*T. aestivum*	Canada	spring	chromosome	[3]
8	CDC Stanley	PANG0004	*T. aestivum*	Canada	spring	chromosome	[3]
9	Chinese Spring	K-44435	*T. aestivum*	China	spring	chromosome	[4]
10	Claire	PANG0005	*T. aestivum*	United Kingdom	winter	scaffold	[3]
11	CM42	—	*T. aestivum*	China	winter	chromosome	[1]
12	Fielder	LPGKU2330	*T. aestivum*	USA	spring	chromosome	[5]
13	HD6172	—	*T. aestivum*	China	winter	chromosome	[1]
14	Jagger	PANG0006	*T. aestivum*	USA	winter	chromosome	[3]
15	JIN50	—	*T. aestivum*	China	winter	chromosome	[6]
16	JM47	—	*T. aestivum*	China	winter	chromosome	[1]
17	Julius	PANG0007	*T. aestivum*	Germany	winter	chromosome	[3]
18	Kenong9204	—	*T. aestivum*	China	winter	chromosome	[7]
19	LongReach Lancer	PANG0009	*T. aestivum*	Australia	spring	chromosome	[3]
20	Mace	PANG0011	*T. aestivum*	Australia	spring	chromosome	[3]
21	MZM	—	*T. aestivum*	China	winter	chromosome	[1]
22	NC4	ZM017424	*T. aestivum*	China	spring	chromosome	[1]
23	Norin 61	PANG0012	*T. aestivum*	Japan	spring	chromosome	[3]
24	Paragon	PANG0013	*T. aestivum*	United Kingdom	spring	scaffold	[3]
25	Renan	PI 564569	*T. aestivum*	France	winter	chromosome	[8]
26	Robigus	PANG0014	*T. aestivum*	United Kingdom	winter	scaffold	[3]
27	S4185	—	*T. aestivum*	China	winter	chromosome	[1]
28	Spelt (PI190962)	PANG0018	*T. spelta*	Italy	winter	chromosome	[3]
29	SY Mattis	PANG0015	*T. aestivum*	France	winter	chromosome	[3]
30	XN6028	ZM009597	*T. aestivum*	China	winter	chromosome	[1]
31	XY6	ZM017079	*T. aestivum*	China	winter	chromosome	[1]
32	YM158	—	*T. aestivum*	China	winter	chromosome	[1]
33	Z8425B	—	*T. aestivum*	China	winter	chromosome	[9]
34	Zang1817	—	*T. aestivum subsp. tibeticum*	China	spring	chromosome	[10]
35	ZM366	—	*T. aestivum*	China	winter	chromosome	[1]

* Accession prefixes: K, VIR (Russia); PI, USDA-NPGS (USA); PANG, JIC SeedStor pangenome collection (UK); ZM, CGRIS (China); LPGKU, Kyoto University/NBRP-Wheat (Japan). The dash means no information available.

** Information on origin and growth habit was obtained from the U.S. Department of Agriculture National Plant Germplasm System, GRIN-Global version 2.3.12 (https://npgsweb.ars-grin.gov/gringlobal/search).

Table S2. Primer pairs used to amplify fragments of wheat *QT12* homeologs for sequencing.

Gene identifier (RefSeq1.0)	Primer name	Primer sequence (5′→3′)	PCR product size, bp
TraesCS3A02G207800	QT12-3A_16ML	TGGATCTCCCCTCTCATTTACCT	1469
	QT12-3A_16R	ATGAACACGGCCTCGCTGAT	
	QT12-3A_48L	GATATAAGGGGCACGCATCACG	1543
	QT12-3A_48R	CCTACATGTAATAAGTACAAGGTTCG	
	QT12-3A_77L	ACAAACTTACTGATGATGCCATTTG	822
	QT12-3A_77R	ACACTCACTGATCTTTCGCAAGT	
	QT12-3A_48L	GATATAAGGGGCACGCATCACG	2252
	QT12-3A_77R	ACACTCACTGATCTTTCGCAAGT	
TraesCS3B02G234900	QT12-3B_19L	ATTGCACAGTGATATTGCGCTAC	1630
	QT12-3B_19R	AACATGGCCTGGCTGAGAAA	
	QT12-3B_132L	CGGCAGGGAATACGTACAGATAA	1839
	QT12-3B_132MR	CCAAAATGTTATCGGTGAAGCAAC	
TraesCS3D02G208200	QT12-3D_16L	CCTAGATAGACGAGTGGGGAGG	1381
	QT12-3D_16R	TCCAAATGCTAAGGTAACCTACA	
	QT12-3D_34L	GGACACTAAAGTTTACTTCTCCTGG	1042
	QT12-3D_34R	TCTTCTCCTGGTTGGTCAGAGTA	
	QT12-3D_16L	CCTAGATAGACGAGTGGGGAGG	2175
	QT12-3D_34R	TCTTCTCCTGGTTGGTCAGAGTA	

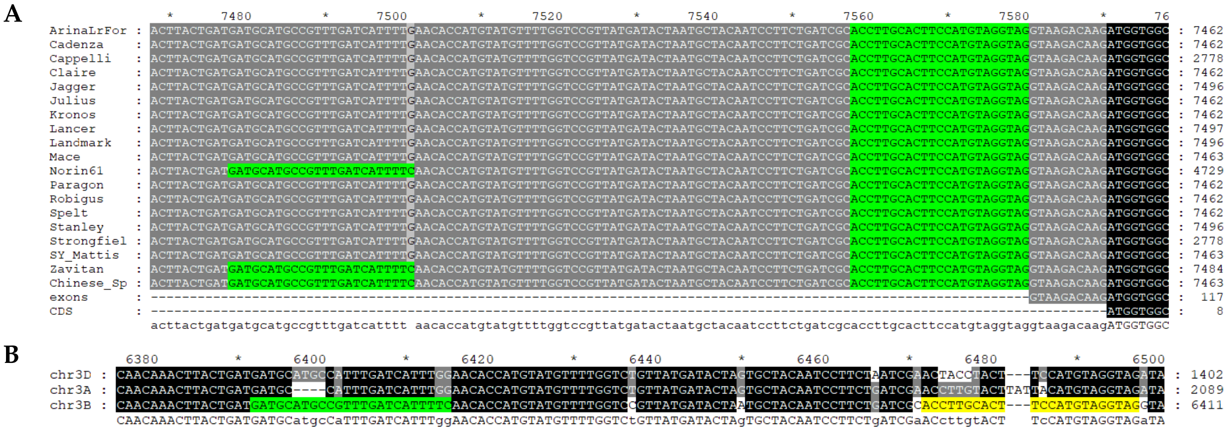

Figure S1. Positions of the KASP-marker primers in an alignment of *TaQT12-B1* sequences from wheat pangenome accessions (A) and in an alignment of the three *QT12* homeologs on chromosomes 3A, 3B, and 3D (B).

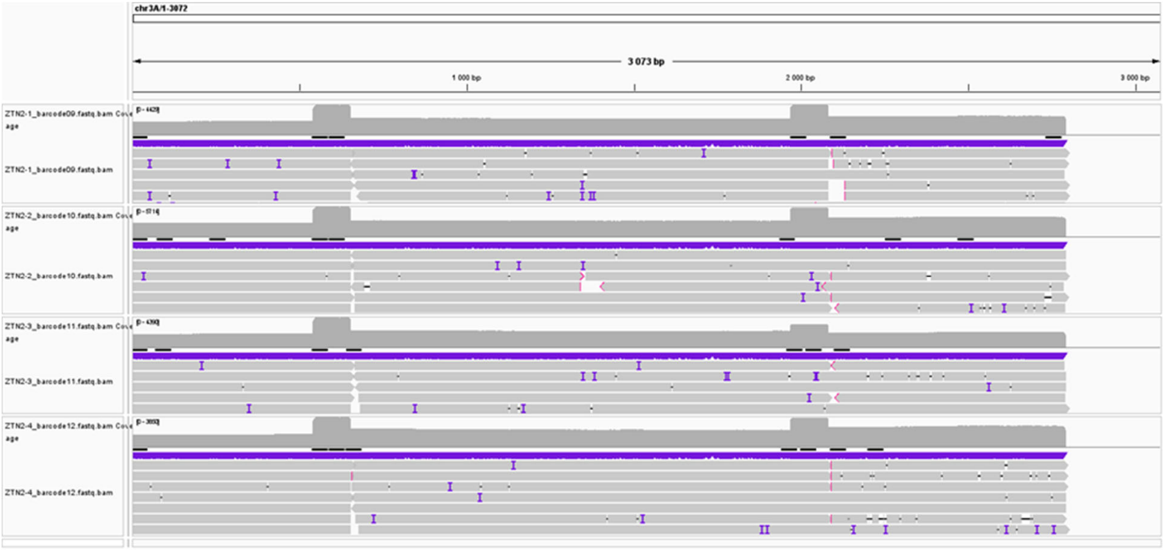

Figure S2. Example alignment of long ONT reads from overlapping PCR amplicons covering *TaQT12-A1* and part of its upstream region. Accessions shown from top to bottom: Grom, Tanya, Shkola, and Mone. Primer sequences and expected amplicon sizes are provided in Table S2, and primer positions are shown in Figure 9.

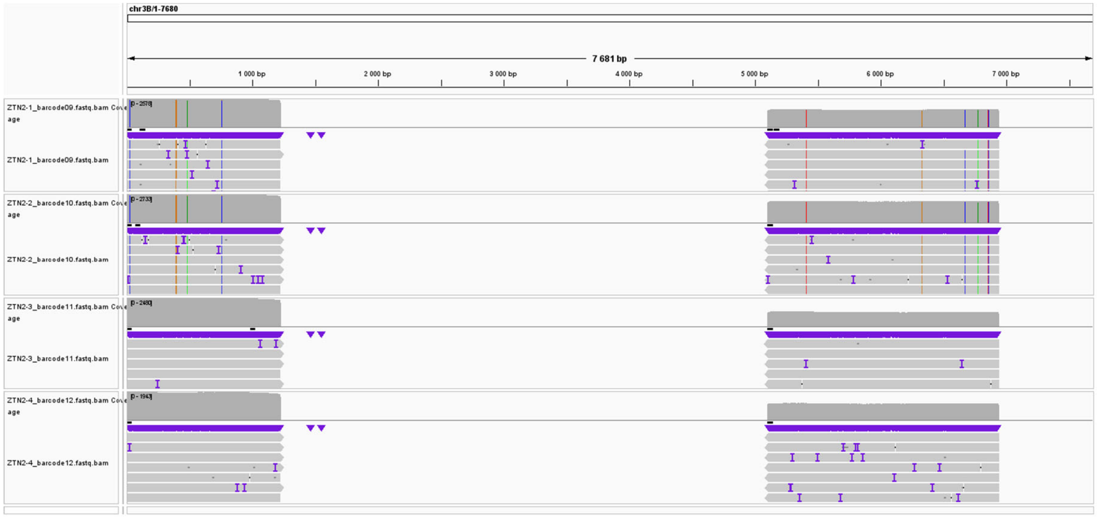

Figure S3. Example alignment of long ONT reads from the two *TaQT12-B1* amplicons in Grom, Tanya, Shkola, and Mone. The amplicons cover both exons and adjacent non-coding regions but do not span the entire long 5′-UTR intron. Primer sequences and expected amplicon sizes are provided in Table S2.

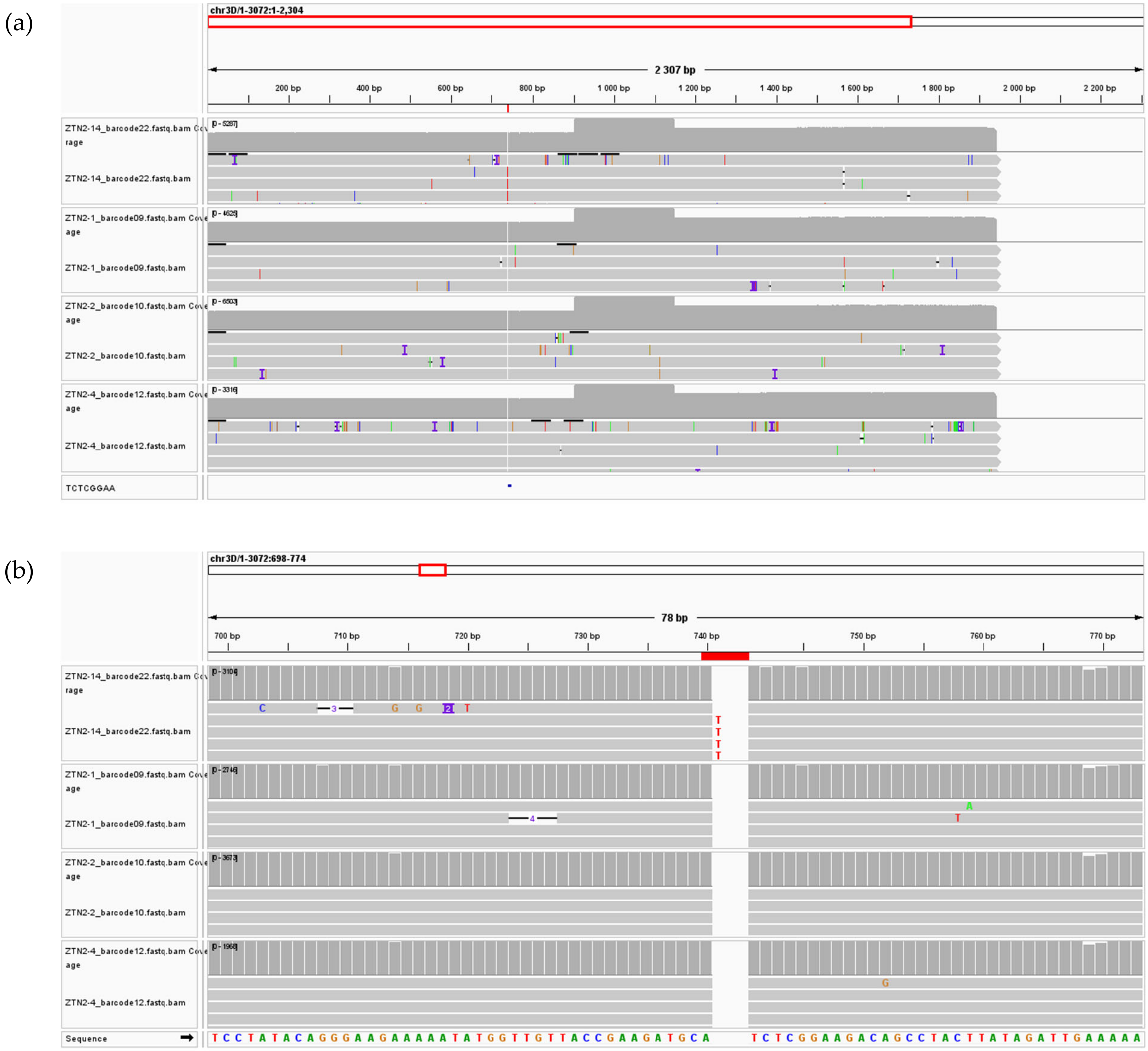

Figure S4. Example alignment of long ONT reads from overlapping *TaQT12-D1* amplicons covering the gene and part of its upstream region. Accessions shown from top to bottom: Likamero (experimental code ZTN2-14), Grom (ZTN2-1), Tanya (ZTN2-2), and Mone (ZTN2-4). (a) Overall alignment; (b) region containing the single-nucleotide insertion detected in the Likamero upstream region.

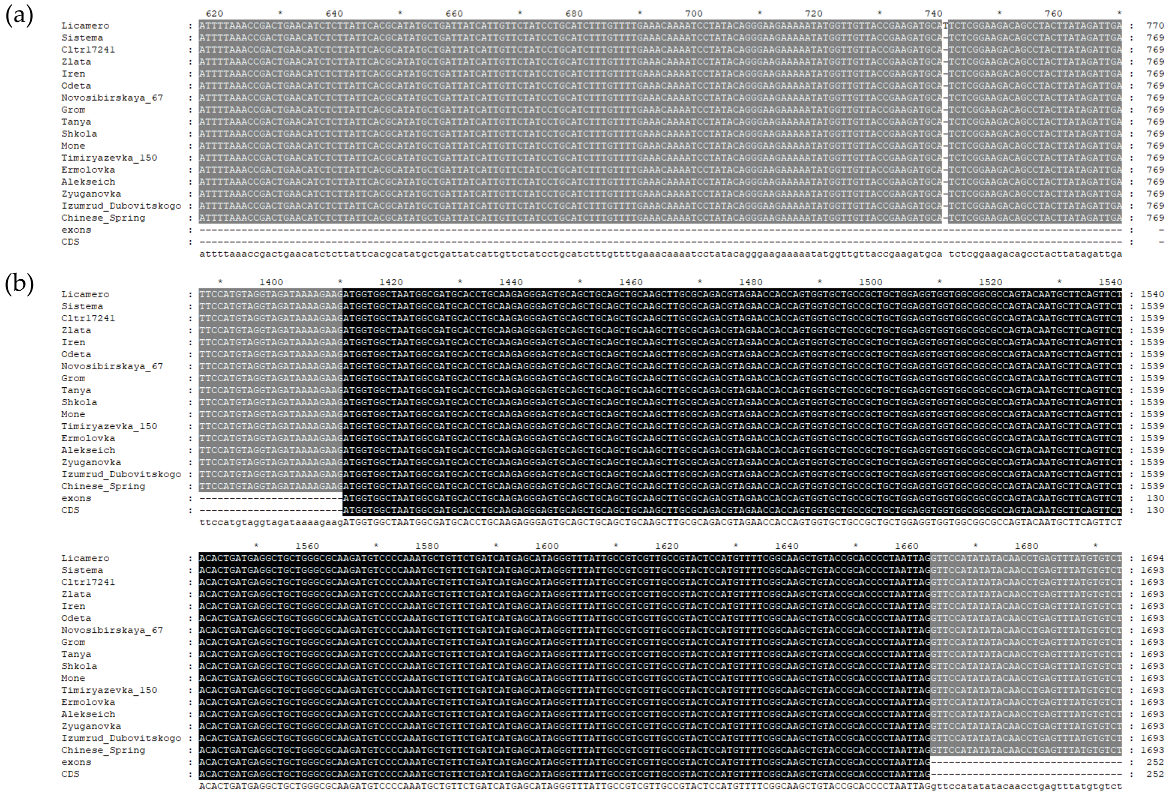

Figure S5. Alignment of *TaQT12-D1* amplicon consensus sequences from Russian and foreign wheat cultivars in the VNIISB collection. (a) The Likamero sequence contains a single-nucleotide insertion 669 bp upstream of the start codon; (b) no polymorphisms were detected in the protein-coding sequence.

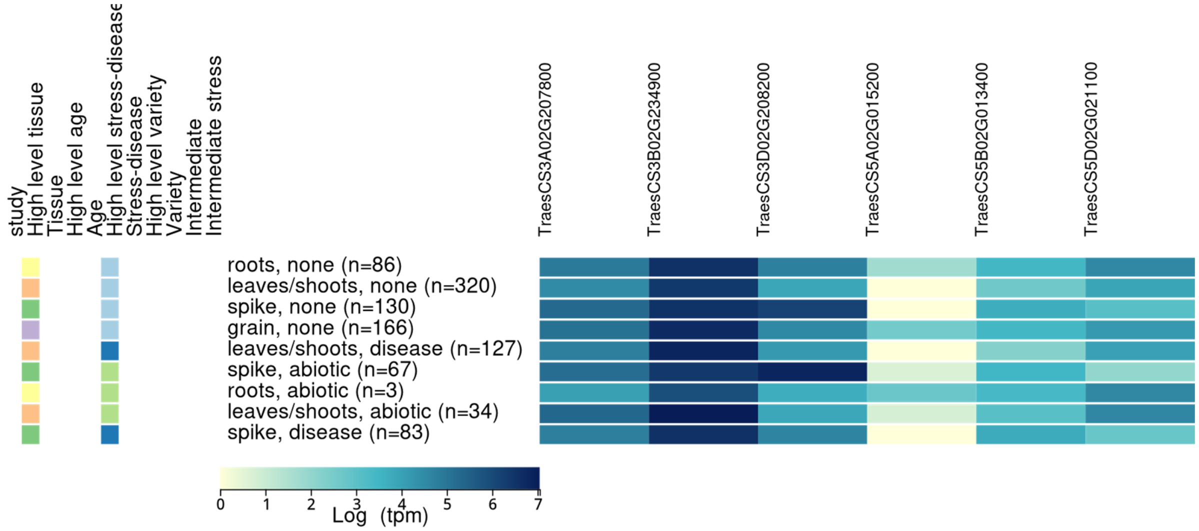

Figure S6. Expression profiles of the six bread wheat co-orthologs of rice *QT12* across aggregated tissue and stress categories in the Wheat Expression Browser (expVIP). Heatmap colors represent log-transformed mean transcript abundance (TPM) for each gene/category combination; the number of samples contributing to each category is shown in the row labels. The chromosome group 3 triad is generally more highly expressed than the chromosome group 5 triad, with *TraesCS3B02G234900* (*TaQT12-B1*) showing the highest mean expression in most categories, including unstressed grain.

Table S3. Mean trait values by year for winter wheat accessions from the P.P. Lukyanenko National Grain Center collection carrying *TaQT12-B1 Hap1* or *Hap2*, with Welch's t-test and FDR-adjusted p-values.

Trait	Year	Mean		Sample size (n)		Welch's t	Raw p-value	FDR-adjusted p-value
		Hap1	***Hap2***	*Hap1*	*Hap2*			
Grain protein content	2018	14.90	13.85	168	4	2.36	0.09	0.40
	2019	15.16	14.78	194	6	1.23	0.27	0.45
	2020	13.60	13.22	177	5	1.35	0.24	0.45
Starch content	2019	65.55	66.67	194	6	-1.96	0.10	0.40
	2020	66.47	67.00	177	5	-1.59	0.18	0.43
Thousand-kernel weight	2018	42.85	38.93	170	4	1.50	0.23	0.45
	2019	35.55	34.03	194	6	1.06	0.33	0.52
	2020	36.74	35.32	177	5	0.94	0.39	0.55
Grain yield	2018	8.52	9.95	170	4	-3.10	0.04	0.40
	2019	7.53	7.87	194	6	-0.59	0.58	0.70
	2020	10.23	11.26	177	5	-1.81	0.14	0.40
Test weight	2018	785.35	776.50	168	4	0.93	0.42	0.55
	2019	736.10	744.50	194	6	-1.65	0.14	0.40
	2020	806.60	818.40	177	5	-1.87	0.12	0.40
Visual grain quality score	2018	6.60	6.75	170	4	-0.20	0.85	0.86
	2019	5.77	5.50	194	6	0.35	0.74	0.84
	2020	6.37	6.60	177	5	-0.19	0.86	0.86

Raw p-values are reported together with Benjamini–Hochberg FDR-adjusted p-values. The Benjamini–Hochberg correction was applied jointly across all 17 trait–year comparisons in this table. FDR-adjusted $p < 0.05$ was considered statistically significant.

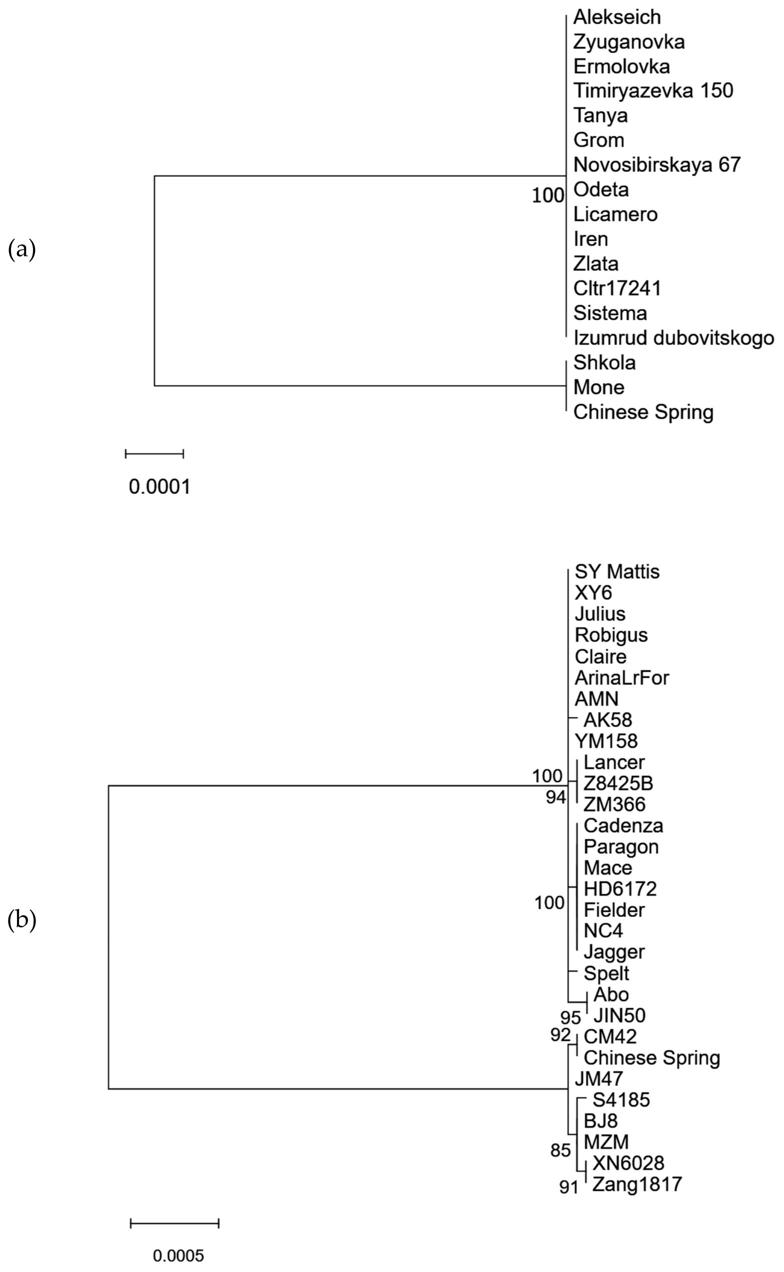

Figure S7. Maximum-likelihood phylogenetic trees of *TaQT12-B1* based on (a) ONT amplicon consensus sequences and the Chinese Spring reference sequence and (b) pangenome-derived sequences. Trees were inferred in MEGA11 using the Tamura–Nei substitution model. Numbers at nodes indicate bootstrap support (%) from 500 replicates. Positions containing gaps or missing data were excluded using the complete-deletion option. Scale bar indicates the genetic distance expressed as an expected number of substitutions per site.

Tables S4–S8 are provided in the accompanying Excel workbook Supplementary_tables_S4-S8.xlsx.

Table S4. Nucleotide variants detected in and around *TaQT12-A1* in the wheat pangenome.

Table S5. Nucleotide variants detected in and around *TaQT12-B1* in the wheat pangenome.

Table S6. Nucleotide variants detected in and around *TaQT12-D1* in the wheat pangenome.

Table S7. Daily maximum air-temperature data for Krasnodar, 2018–2020.

Table S8. Phenotype data for winter wheat accessions evaluated at the P.P. Lukyanenko National Grain Center, 2018–2020. In the Genotype_QT12 column, 0 denotes Hap1 (G/HEX) and 2 denotes Hap2 (C/FAM); NaN denotes missing phenotypic data. Accession prefix: K, VIR (Russia).

Supplementary sequence data are provided in the accompanying archive and include pangenome-derived and ONT consensus FASTA files for *TaQT12-A1*, *TaQT12-B1*, and *TaQT12-D1*.

Table S9. Mean and minimum–maximum ONT read depth across primer-defined target regions (×; mean with minimum–maximum in parentheses)

Accession	*TaQT12-A1*	*TaQT12-B1*	*TaQT12-D1*
Grom	2,570.0 (1,693–4,429)	1,977.5 (63–2,578)	3,210.4 (2,352–4,625)
Tanya	3,344.0 (2,259–5,714)	2,312.5 (69–2,733)	4,427.1 (3,194–6,503)
Shkola	2,386.1 (1,707–4,390)	1,729.1 (980–2,480)	2,522.3 (1,759–3,645)
Mone	2,336.2 (1,359–3,850)	1,435.2 (870–1,943)	2,348.9 (1,682–3,316)
Timiryazevka 150	3,698.6 (2,587–6,132)	2,510.8 (73–3,203)	4,315.9 (3,077–6,208)
Ermolovka	5,053.1 (3,071–8,403)	3,073.3 (108–3,202)	5,160.9 (3,637–7,529)
Alekseich	2,796.5 (1,912–4,824)	1,809.4 (51–2,456)	3,137.2 (2,277–4,459)
Zyuganovka	3,240.2 (1,984–5,417)	2,093.0 (58–2,602)	3,348.8 (2,287–4,820)
Izumrud Dubovitskogo	3,458.4 (2,255–5,986)	2,076.7 (58–2,628)	3,048.0 (2,172–4,376)
Sistema	4,398.3 (2,129–7,232)	2,748.8 (99–2,962)	4,687.2 (3,402–6,934)
CItr 17241 (Chris Mutant)	2,313.1 (1,569–4,067)	1,255.4 (26–1,934)	2,209.2 (1,665–3,159)
Zlata	2,998.9 (1,839–5,050)	1,718.7 (48–2,261)	2,776.7 (1,980–4,019)
Iren	3,894.2 (2,024–6,498)	2,616.5 (103–2,786)	4,895.3 (3,575–7,243)
Likamero	3,399.7 (1,754–5,555)	2,169.8 (66–2,469)	3,655.3 (2,677–5,287)
Odeta	4,058.0 (2,334–6,837)	2,572.7 (91–2,712)	3,744.6 (2,702–5,484)
Novosibirskaya 67	2,854.0 (1,817–4,822)	1,610.3 (54–2,202)	2,768.0 (1,961–3,944)

Depth values come from the original samtools depth output. Consequently, local depth minima can coincide with a deletion rather than indicate a lack of reads spanning that position.

Table S10. Percentages of target positions below half the accession-specific mean depth

Accession	*TaQT12-A1*	*TaQT12-B1*	*TaQT12-D1*
Grom	0.000	0.033	0.000
Tanya	0.000	0.033	0.000
Shkola	0.000	0.000	0.000
Mone	0.000	0.000	0.000
Timiryazevka 150	0.000	0.033	0.000
Ermolovka	0.000	0.033	0.000
Alekseich	0.000	0.033	0.000
Zyuganovka	0.000	0.033	0.000
Izumrud Dubovitskogo	0.000	0.033	0.000
Sistema	0.036	0.033	0.000
CItr 17241 (Chris Mutant)	0.000	0.033	0.000
Zlata	0.000	0.033	0.000
Iren	0.000	0.033	0.000
Likamero	0.000	0.033	0.000
Odeta	0.000	0.033	0.000
Novosibirskaya 67	0.000	0.033	0.000

Values are percentages (%). For each sample and locus, μ = sum of depths/N, where N is the number of target positions. The low-depth fraction is 100 × number of positions with depth < $\mu/2$ divided by N.

Table S11. Comparison of ONT amplicon sequencing and the *TaQT12-B1* KASP marker for the six wheat accessions assessed by both methods. The marker targets the 5′-UTR intron SNP c.-10-80C>G.

Accession	ONT SNP allele	ONT sequence group	KASP result[1]	Agreement[2]
Alekseich	G	*Hap1*	G / HEX (Hap1)	Yes
Grom	G	*Hap1*	G / HEX (Hap1)	Yes
Mone	C	*Hap2*	C / FAM (Hap2)	Yes
Shkola	C	*Hap2*	C / FAM (Hap2)	Yes
Tanya	G	*Hap1*	G / HEX (Hap1)	Yes
Timiryazevka 150	G	*Hap1*	G / HEX (Hap1)	Yes

All six accessions showed concordant results (6/6; 100%), comprising four G/HEX accessions associated with Hap1 and two C/FAM accessions associated with *Hap2*.

[1] The C allele detected by FAM corresponds to the *Hap2*-associated state, whereas the G allele detected by HEX corresponds to the *Hap1*-associated state.

[2] Agreement denotes correspondence between the ONT-derived SNP allele and the KASP allele call.

References:

1. Jiao, C.; Xie, X.; Hao, C.; Chen, L.; Xie, Y.; Garg, V.; Zhao, L.; Wang, Z.; Zhang, Y.; Li, T.; et al. Pan-Genome Bridges Wheat Structural Variations with Habitat and Breeding. *Nature* **2025**, *637*, 384–393, doi:10.1038/s41586-024-08277-0.
2. Jia, J.; Zhao, G.; Li, D.; Wang, K.; Kong, C.; Deng, P.; Yan, X.; Zhang, X.; Lu, Z.; Xu, S.; et al. Genome Resources for the Elite Bread Wheat Cultivar Aikang 58 and Mining of Elite Homeologous Haplotypes for Accelerating Wheat Improvement. *Mol. Plant* **2023**, *16*, 1893–1910, doi:10.1016/j.molp.2023.10.015.
3. Walkowiak, S.; Gao, L.; Monat, C.; Haberer, G.; Kassa, M.T.; Brinton, J.; Ramirez-Gonzalez, R.H.; Kolodziej, M.C.; Delorean, E.; Thambugala, D.; et al. Multiple Wheat Genomes Reveal Global Variation in Modern Breeding. *Nature* **2020**, *588*, 277–283, doi:10.1038/s41586-020-2961-x.
4. The International Wheat Genome Sequencing Consortium (IWGSC); Appels, R.; Eversole, K.; Stein, N.; Feuillet, C.; Keller, B.; Rogers, J.; Pozniak, C.J.; Choulet, F.; Distelfeld, A.; et al. Shifting the Limits in Wheat Research and Breeding Using a Fully Annotated Reference Genome. *Science* **2018**, *361*, eaar7191, doi:10.1126/science.aar7191.
5. Sato, K.; Abe, F.; Mascher, M.; Haberer, G.; Gundlach, H.; Spannagl, M.; Shirasawa, K.; Isobe, S. Chromosome-Scale Genome Assembly of the Transformation-Amenable Common Wheat Cultivar 'Fielder.' *DNA Res.* **2021**, *28*, dsab008, doi:10.1093/dnares/dsab008.
6. Lin, J.; Zhang, C.; Liu, Z.; Li, J.; Yang, Q.; Chu, W.; Liu, D.; Zhang, L.; Zhao, D.; Peng, X.; et al. Genomic and Genetic Dissection of Drought Tolerance in a Resilient Wheat Germplasm JIN50. *Nat. Genet.* **2026**, doi:10.1038/s41588-026-02596-w.
7. Shi, X.; Cui, F.; Han, X.; He, Y.; Zhao, L.; Zhang, N.; Zhang, H.; Zhu, H.; Liu, Z.; Ma, B.; et al. Comparative Genomic and Transcriptomic Analyses Uncover the Molecular Basis of High Nitrogen-Use Efficiency in the Wheat Cultivar Kenong 9204. *Mol. Plant* **2022**, *15*, 1440–1456, doi:10.1016/j.molp.2022.07.008.
8. Aury, J.-M.; Engelen, S.; Istace, B.; Monat, C.; Lasserre-Zuber, P.; Belser, C.; Cruaud, C.; Rimbert, H.; Leroy, P.; Arribat, S.; et al. Long-Read and Chromosome-Scale Assembly of the Hexaploid Wheat Genome Achieves High Resolution for Research and Breeding. *GigaScience* **2022**, *11*, giac034, doi:10.1093/gigascience/giac034.
9. Li, G.; Ren, Y.; Yang, Y.; Chen, S.; Zheng, J.; Zhang, X.; Li, J.; Chen, M.; Sun, X.; Lv, C.; et al. Genomic Analysis of Zhou8425B, a Key Founder Parent, Reveals Its Genetic Contributions to Elite Agronomic Traits in Wheat Breeding. *Plant Commun.* **2025**, *6*, 101222, doi:10.1016/j.xplc.2024.101222.
10. Guo, W.; Xin, M.; Wang, Z.; Yao, Y.; Hu, Z.; Song, W.; Yu, K.; Chen, Y.; Wang, X.; Guan, P.; et al. Origin and Adaptation to High Altitude of Tibetan Semi-Wild Wheat. *Nat. Commun.* **2020**, *11*, 5085, doi:10.1038/s41467-020-18738-5.
